# Magnetic field platform for experiments on well-mixed and spatially structured microbial populations

**DOI:** 10.1016/j.bpr.2024.100165

**Published:** 2024-06-17

**Authors:** Akila Bandara, Enoki Li, Daniel A. Charlebois

**Affiliations:** 1 Department of Physics, University of Alberta, Edmonton, Alberta, Canada; 2 Department of Biomedical Engineering, University of Alberta, Edmonton, Alberta, Canada

## Abstract

Magnetic fields have been shown to affect sensing, migration, and navigation in living organisms. However, the effects of magnetic fields on microorganisms largely remain to be elucidated. We develop an open-source, 3D-printed magnetic field exposure device to perform experiments on well-mixed and spatially structured microbial populations. This device is designed in AutoCAD, modeled in COMSOL, and validated using a Gaussmeter and experiments on the budding yeast *Saccharomyces cerevisiae*. We find that static magnetic field exposure slows the spatially structured expansion of yeast mats that expand in two dimensions, but not yeast mats that expand in three dimensions, across the surface of semi-solid yeast extract-peptone-dextrose agar media. We also find that magnetic fields do not affect the growth of planktonic yeast cells in well-mixed liquid yeast extract-peptone-dextrose media. This study provides an adaptable device for performing controlled magnetic field experiments on microbes and advances our understanding of the effects of magnetic fields on fungi.

## Why it matters?

Microorganisms have been shown to be affected by magnetic fields. However, the effects of magnetic fields on microbial populations are largely unknown. This is especially true for fungi, which are important microorganisms for microbiological research, industrial applications, and infectious disease. To study magnetobiological phenomena, we need devices to perform controlled experiments in a variety of conditions. We develop an open-source, 3D-printed magnetic field platform using computer-aided design and physics modeling software to study the effects of magnetic fields on microbial populations. Using this magnetic field device, we find that magnetic fields can slow the growth of budding yeast on agar plates but that magnetic fields do not affect the growth of budding yeast in liquid media.

## Introduction

The effect of electromagnetic fields (EMFs) on the adaptation and evolution of life on Earth is an ongoing area of research (1). Living organisms including sharks, bees, and birds use EMFs to sense, navigate, and migrate (2). Magnetic fields (MFs) have been shown to affect the germination of plants (3) and the orientation of blood cells (4,5) and alter stem cell-mediated growth of flatworms (6). Magnetotactic bacteria can align with external MFs by biomineralizing magnetic nanoparticles (magnetite or greigite) inside organelles called magnetosomes, which is thought to aid bacteria to reach regions of optimal oxygen concentration (7). Magnetic nanoparticles have been implemented in cell labeling and imaging (8), as well as targeted drug delivery applications (9). Despite the advantages of EMFs for living microorganisms, it is important to investigate the detrimental effects of EMF exposure (10).

Due to a short replication time, ease of culture, and well-characterized eukaryotic genetic background, the budding yeast *Saccharomyces cerevisiae* is a ubiquitous model organism in molecular biology (11). *S. cerevisiae* has been used as a model organism in EMF and MF exposure studies (12,13,14,15,16,17). For instance, using a single-cell MF device, the orientation of individual *S. cerevisiae* cells was found to align with a static MF during budding (12). Exposure to a strong 50,000 *G* vertical MF resulted in changes in the sedimentation pattern of yeast cells, depending on their location in the culture dishes (14). However, in the same study, no changes in gene expression were observed after exposure to a 50,000 *G* MF (for 2 and 24 h) or to a 100,000 *G* MF (for 1 h).

Previous studies highlight the necessity to study the isolated effects of MFs on fungi. For example, exposing plant pathogenic fungi to low-frequency EMFs and MFs separately yields diverging results: EMFs (1G at 50Hz) were found to have no effect on the growth of mycorrhizal fungi (18), whereas MFs (1−10G) slowed the growth of phytopathogenic fungi (17). Another study found that exposing nonpathogenic *S. cerevisiae* cells to stronger EMFs (50 Hz with inductions up to 10mT, with exposure times up to 24 min) decreased the viability of yeast cells and slowed their growth (19). More recently, a 2D lattice-based Monte Carlo simulation framework was developed to investigate the effects of nutrient concentration and MF exposure on yeast colony growth and morphology (20). Simulation of this framework predicted that prolonged MF exposure will decrease colony growth and alter colony morphology. Despite this research, our knowledge of the effects of MFs on fungi remains limited (17). Furthermore, though there are many studies on organisms exposed to EMFs (e.g., (19,21,22,23,24)), the effects of static MF on the growth and development of microorganisms largely remain to be investigated (12).

Yeast exist as part of microbial communities, including colonies, biofilms (25), and “mats” (26,27,28,29,30). A yeast mat is a morphologically complex, colony-like structure that requires the expression of the flocculin gene *flo11*, which encodes the *Flo11* surface adhesion protein. Chen et al. (31) cultured strains of genetically engineered *S. cerevisiae* cells with and without the *flo11* gene, called TBR1 and TBR5, respectively. “Wild-type” TBR1 mats (formed from TBR1 cells with a functional copy of *flo11*) displayed a rough surface, pattern-forming phenotype, whereas mutant TBR5 mats (formed from cells lacking a functional copy of *flo11*) displayed a smooth surface phenotype. TBR1 mats grew faster and larger compared to TBR5 mats on semi-solid agar plates; TBR1 also outcompeted TBR5 in competition assays. TBR1 mats are constrained to expand in 2D along the agar surface due to the expression of *Flo11* in TBR1 cells. As a result, TBR1 mats had a higher growth rate (fitness) than TBR5 mats, which expand in three dimensions, resulting in a slower mat expansion rate and size along the agar surface. In contrast, there was no difference in fitness between TBR1 and TBR5 strains in liquid culture experiments (31). Other studies have found similar results for TBR1 and TBR5 growth rates in semi-solid and liquid media cultures (26,32). Given the discernible phenotypes and differential growth rates of the TBR1 and TBR5 strains on agar media, this model system is ideal to investigate the effects of MF exposure on population growth dynamics and generate hypotheses on the underlying biophysical mechanisms.

In this study, we develop an adaptable, open-source MF exposure platform to investigate of the effects of MF on microbial population over extended periods. The device consists of two neodymium (Nd_2_Fe_14_B) magnets that expose multiple biological replicates to a static MF in the range of 350 to 1500 *G*. The compact design of the MF device allows it to be placed inside of an incubator or environmental chamber to perform controlled experiments. We design the MF exposure device in AutoCAD (33) and optimize the design via numerical simulations in COMSOL Multiphysics (34). We then 3D print the optimized device and measure the MF inside the sample exposure region, which is compared to numerical COMSOL simulations. To highlight the utility of our device, we perform MF experiments on well-mixed and spatially structured yeast populations inside of an environmental chamber. We find that 1) horizontal and vertical MFs slow the growth of TBR1 mats on semi-solid agar media but MFs do not affect the growth of TBR5 mats, 2) TBR1 and TBR5 mats adapt over the duration of the experiment to increase their growth rates on agar media in both the presence and absence of MFs, and 3) MFs do not affect the growth rate of planktonic TBR1 and TBR5 cells in liquid media.

## Materials and methods

### Fabricating the MF exposure device

Previous experiments established that a range of magnetic flux densities ($\vec{B}$) affect the growth or the orientation of yeast cells, ranging from 100 (19) to 29,300 *G* (12). Motivated by these studies, we developed an adaptable, open-source platform capable of generating horizontal or vertical homogeneous MFs within this range. This MF device was designed to perform controlled and repeatable long-term, population-scale magnetobiological experiments on microorganisms. As such, the MF device was optimized to be placed in an incubator/environment chamber while having the capacity to accommodate multiple biological replicates of microbes cultured in standard-size culture tubes and Petri dishes. The AutoCAD, COMSOL, and 3D printing files are freely available online (see supporting material) to facilitate the replication and adaptation of our platform by other research groups for their magnetobiological experiments.

**Figure 1:**
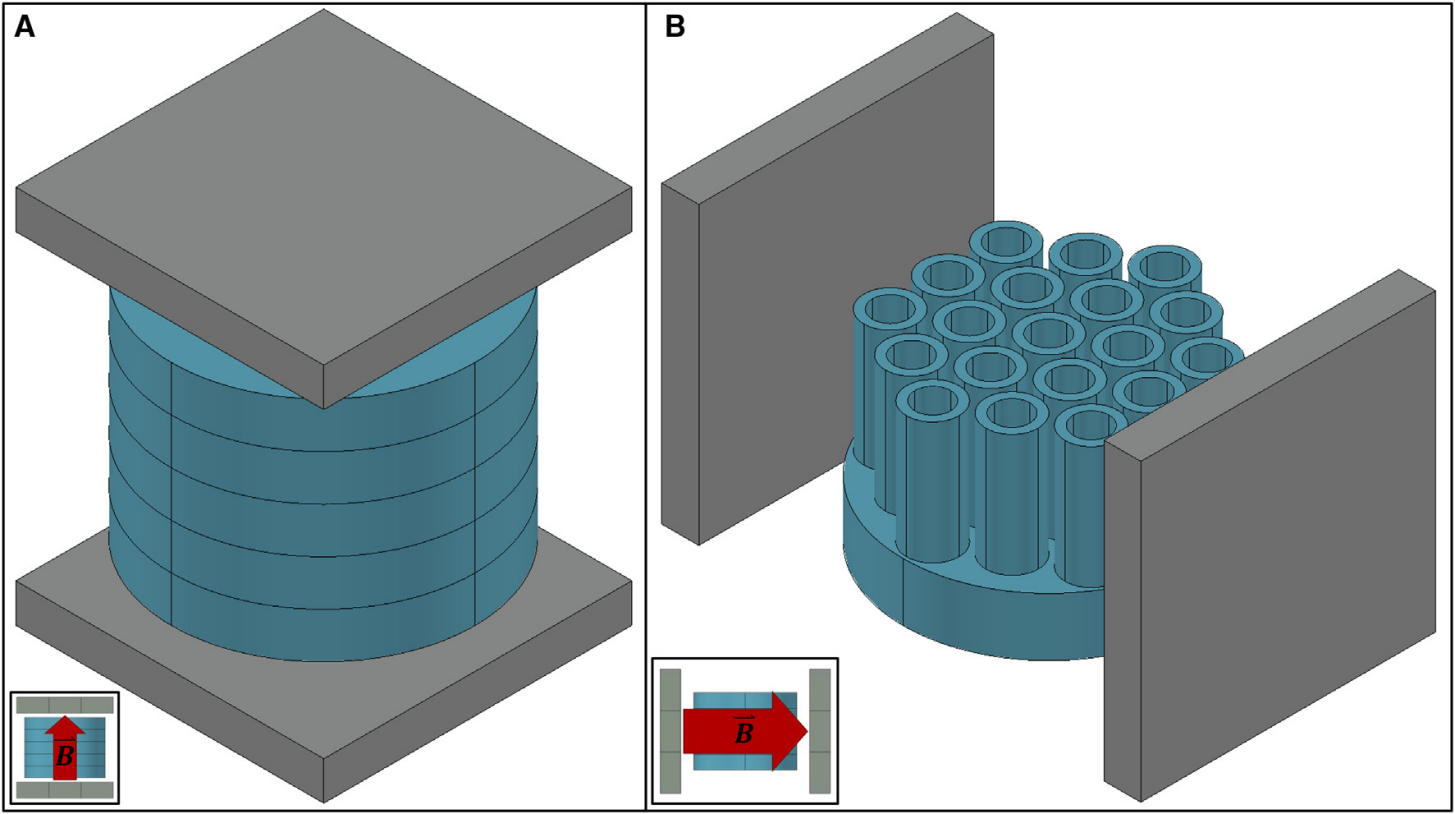
AutoCAD (33) schematics showing some different configurations of the MF device. (*A*) Schematic of the vertical configuration of the device, which was used to expose yeast grown on semi-solid media in Petri dishes to a static vertical magnetic field (MF). Petri dishes are shown in blue. (*B*) Schematic of the horizontal configuration of the device, which was used to expose yeast grown in liquid media in cell culture tubes to a static horizontal MF. Culture tubes and holder are shown in blue. The gray blocks in (*A*) and (*B*) represent the neodymium magnets; insets show the direction of the magnetic flux density ($\vec{B}$).

To create a homogeneous MF to expose biological samples of microbes growing in cell culture tubes or Petri dishes, we used two N52-grade Nd_2_Fe_14_B block magnets (Amazing Magnets, Round Rock, TX, USA, catalog no. Q500Y-N52). The device’s ability to hold multiple replicates (five Petri dishes or 19 culture tubes) for each exposure experiment is essential to ensure the repeatability of the results and test for statistical significance. The horizontal and vertical configurations of the block magnets in the device permit MF exposure emanating from two different directions (Figs. 1, *A* and *B*, and S1). With dimensions of 101.6×101.6×12.7 $mm^{3}$, these magnets were capable of producing a $\vec{B}$ of approximately 14,400 *G* at the magnet’s surface, which is five orders of magnitude stronger than the Earth’s MF (0.24*–*0.66 *G* depending on the latitude (35)).

The MF exposure device materials were selected to minimize interaction between the magnets and the scaffolding. Considering that microorganisms will typically be grown on semi-solid media in Petri dishes or liquid media in culture tubes (see yeast MF exposure experiments), it was necessary to fabricate the MF device using a material that had compatible magnetic permeability (*μ*) to the Petri dishes and culture tubes. This ensures that the homogeneous MFs produced by the block magnets would have the least possible interference during its path toward the exposure samples (36). Since two strong magnets are in close proximity (132 mm), the device materials need to be sufficiently strong to withstand a pull force of 5.78 N without compromising the structural integrity of the device. Furthermore, as the device is intended to perform experiments on biological samples, it was designed to be disassembled so that the components can be cleaned and sterilized.

**Figure 2:**
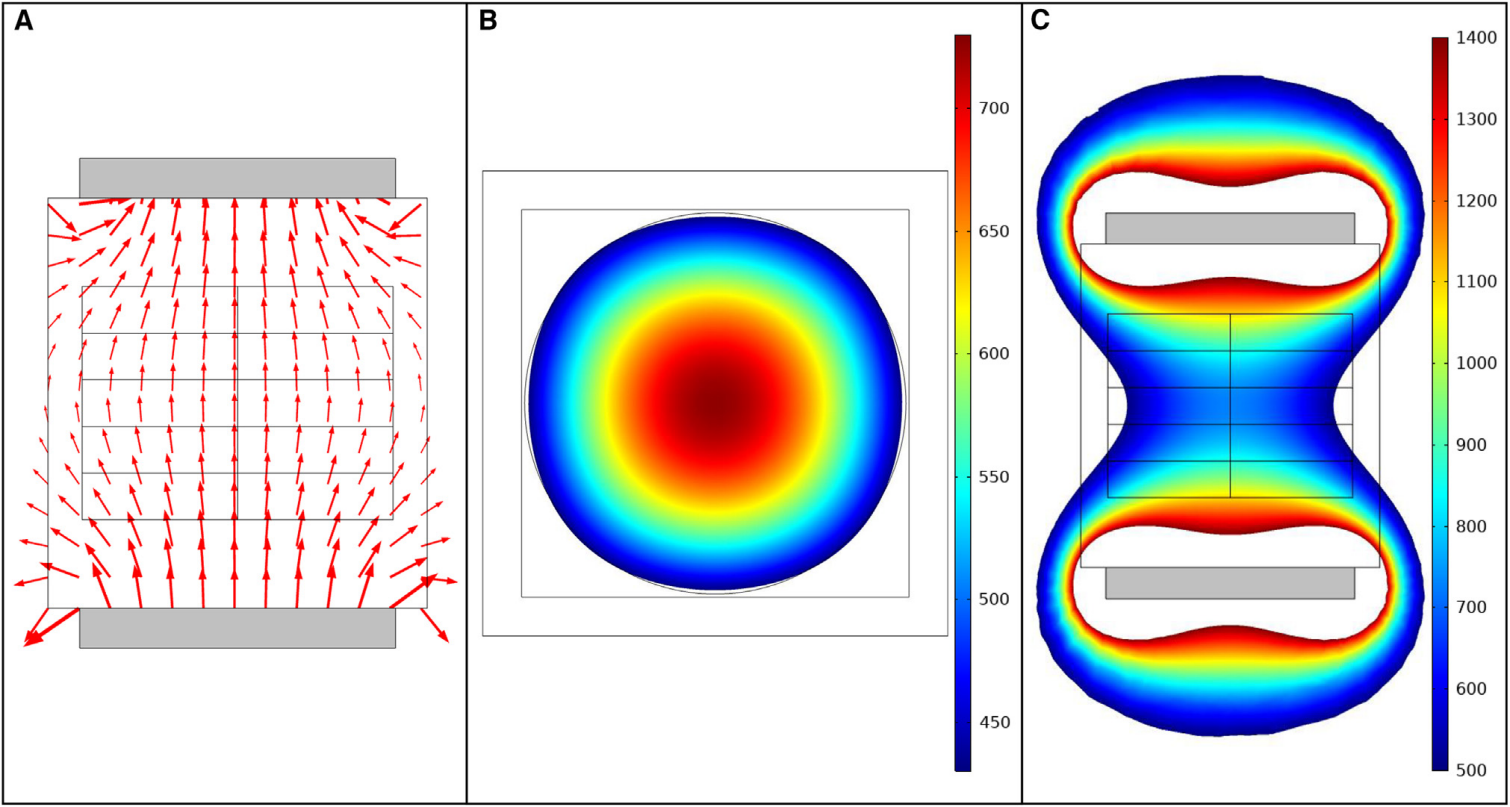
Simulation of the vertical configuration of the MF device. (*A*) COMSOL (34) simulation of the directional orientation of the MF. (*B*) COMSOL simulation of the $\vec{B}$ along the central horizontal plane of the exposure device. The point of view of this image is from above, looking down at the central Petri dish. (*C*) COMSOL simulation of the $\vec{B}$ along the central vertical plane of the exposure device. The point of view of this image is from the side of the exposure device, looking at the sides of the Petri dishes. The gray blocks in (*A*) and (*C*) denote the neodymium magnets. The color bars denote $\vec{B}$ in Gauss (*G*).

**Figure 3:**
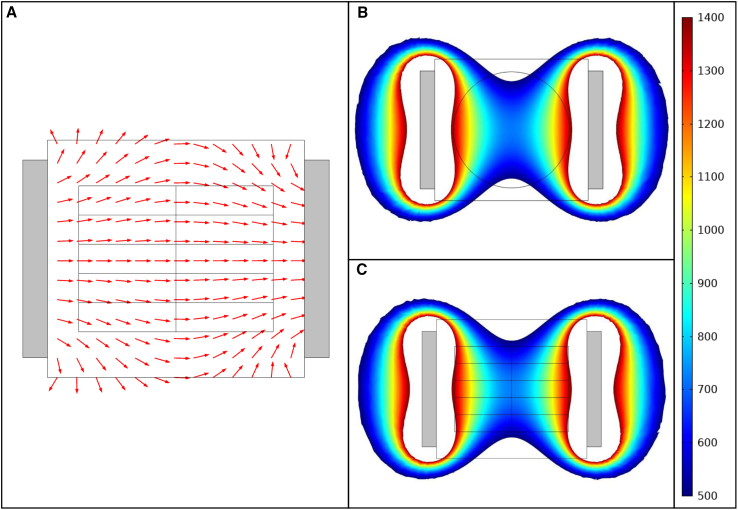
Simulation of the horizontal configuration of the MF device. (*A*) COMSOL (34) simulation results of the directional orientation of the MF. (*B*) COMSOL simulation of the $\vec{B}$ along the central horizontal plane of the exposure device. The point of view of this image is from above, looking down at the central Petri dish. (*C*) COMSOL simulation of the $\vec{B}$ along the central plane from the side view of the exposure device. The gray blocks in (*A*)–(*C*) represent the neodymium magnets. The color bar denotes $\vec{B}$ in *G*.

A cross-sectional area of 101.6×101.6 $mm^{2}$ ensured that the magnets generated a homogeneous MF throughout the sample exposure region. While the magnitude of MF strength varied spatially throughout the exposure region, the direction of the field is almost uniform (Figs. 2 *A* and 3 *A*), and therefore no “edge effects” were introduced in our MF exposure experiments. This assured that the yeast colonies grown in both liquid and semi-solid media were exposed only to homogeneous MF, thus avoiding MF edge effects.

We used AutoCAD (33) to design a MF device with two interchangeable configurations: a horizontal $\vec{B}$ configuration and a vertical $\vec{B}$ configuration (see Fig. S1). COMSOL Multiphysics (34) was used to simulate the MF inside the device to optimize the design (see MF device optimization and validation). The “magnetic fields, no currents” interface in the AC/DC module of COMSOL was used to simulate the $\vec{B}$ values within exposure region of the MF device via a finite element method (37). The MF strength ($\vec{H}$) and the $\vec{B}$ resulting from the permanent magnets in the exposure device are, respectively, described by Maxwell’s equations for a static MF (38):

(1) $$\nabla \times \vec{H}=0$$

and

(2) $$\nabla \cdot \vec{B}=0\text{.}$$

From Eq. 1, we can define the magnetic scalar potential ($V_{m}$) as

(3) $$\vec{H}=-\nabla V_{m}\text{,}$$

and considering the relation between the $\vec{B}$ and $\vec{H}$ yields

(4) $$\vec{B}=\mu_{0}\mu_{rec}\vec{H}+{\vec{B}}_{r}\text{,}$$

where $\mu_{0}$, $\mu_{r}$, and ${\vec{B}}_{r}$ represent the permeability of free space, the recoil permeability, and the remnant flux density of the permanent magnet, respectively. By combining Eqs. 3 and 4 into 2, we obtain

(5) $$-\nabla \cdot \left(\mu_{0}\mu_{rec}\nabla V_{m}-{\vec{B}}_{r}\right)=0\text{.}$$

Eq. 5 was used in our numerical COMSOL simulations to describe the material properties of the Nd_2_Fe_14_B block magnets. The *μ* of polylactic acid (PLA) was also incorporated into the COMSOL simulations to specify the device material.

We used PLA (RepRap Warehouse, Edmonton, Alberta, Canada, catalog no. R00100002), a thermoplastic polyester with a low melting point, high strength, and low thermal expansion, and a *μ* comparable to the Petri dishes/culture tubes to provide durability and versatility to our MF exposure device. The components of the device were 3D printed using PLA filament with a diameter of 1.75 mm with Prusa i3 MK3S and i3 MK3S+ printers.

### Exposure device MF simulations and measurements

COMSOL Multiphysics (34) was used to simulate the MFs of the prototype devices to optimize their design before they were 3D printed. The AutoCAD (33) designs of the device were imported into COMSOL, and the properties of each material used in the device were defined based on their value of *μ* (see Table S1).

The $\vec{B}$ in the exposure region of the horizontal MF exposure device was experimentally measured using a Gaussmeter (AlphaLab, Pittsburgh, PA, USA, Model GM2 Gaussmeter), which operates based on the Hall effect (39). Considering that the two neodymium magnets were kept 132 mm apart from each other in the horizontal and vertical MF exposure devices, the MFs generated in both devices are identical. However, depending on the horizontal or vertical configuration of the MF device, the yeast mats grown in Petri dishes experienced different MF strengths. Although the $\vec{B}$ in the exposure region of the assembled horizontal MF configuration was straightforward to measure, the exposure region of the vertical MF device was difficult to access with the Gaussmeter probe when the device was assembled. Therefore, we only measured the $\vec{B}$ for the horizontal configuration of the exposure device. To create this $\vec{B}$ map, a custom cylindrical Gaussmeter probe holder containing 83 equally spaced rectangular holes was designed and 3D printed (Fig. S2 *A*). This permitted us to measure the $\vec{B}$ in each layer at specific positions inside the exposure chamber (Fig. S2 *B*). We were able to limit the $\vec{B}$ mapping to three layers due to the symmetry of the MF. Four $\vec{B}$ readings were obtained for each position in the these layers. The average of these four readings was used to obtain the final mapping of $\vec{B}$.

### Yeast MF exposure experiments

Haploid *S. cerevisiae* TBR1 (Σ 1278b, matα, *flo11*, tryp) and TBR5 (Σ 1278b, matα, *flo11* Δ, tryp) strains were used for the MF exposure experiments (31). TBR1 (*flo11*) and TBR5 (*flo11* Δ) cells are isogenic apart from the presence or absence of the *flo11* gene, respectively.

Biological replicates of TBR1 and TBR5 were cultured from isogenic colonies in yeast extract-peptone-dextrose (YPD) liquid medium at 30°C and shaken overnight on a CO_2_-resistant shaker (Thermo Scientific, Waltham, MA, USA, catalog no. 88-881-103) at 150 rpm inside an environmental chamber (Thermo Scientific, catalog no. 13-067-066). YPD media were made with 5 g yeast extract (Sigma-Aldrich, St. Louis, MO, USA, catalog no. Y1625), 10 g Bacto peptone (BD, Franklin Lakes, NJ, USA, catalog no. 211677), 38 mg adenine (Sigma-Aldrich, catalog no. D16), and 7.5 g (or a 1.5% final concentration) agar (for agar plates: Fisher Scientific, Hampton, NH, USA, catalog no. BP1423), autoclaved in 450 mL type 1 water, and supplemented with glucose (Fisher Scientific, catalog no. D16) to a final concentration of 2%. Liquid TBR1 and TBR5 cultures were inoculated to $5.5\times 10^{5}$ cells/mL in 5 mL culture tubes (Fisher Scientific, catalog no. 22-171-606). Agar plates were made by pouring YPD agar medium into a “100 mm” (outer diameter is ≈92mm and inner diameter is ≈88mm) Petri dish (Fisher Brand, catalog no. FB0875712) until it was approximately 2/3 full (to minimize agar evaporation and nutrient depletion effects during the experiments). To investigate the effect of MF exposure on spatially structured yeast populations, YPD agar plates were inoculated with a 2 *μ*L drop of $10^{7}$ cells/mL of either the TBR1 or TBR5 strain in the center of the agar. Six control (no-MF) replicates and six experimental (MF) replicates were used for each experiment; in some experiments, a replicate(s) had to be discarded due to contamination or insufficient agar medium (n≠6 in these cases).

For the agar culture horizontal and vertical MF exposure experiments, twenty 100 mm Petri dishes (half-seeded with TBR1 cells and half-seeded with TBR5 cells) were placed inside of four 3D-printed devices. Five TBR1 Petri dishes and five TBR5 Petri dishes were place inside two separate exposure devices (block magnets present), and five TBR1 plates and five TBR5 plates were placed inside two separate control devices (block magnets absent). The MF exposure devices were set up in either the vertical configuration (Fig. 1 *A*) or the horizontal configuration (Fig. 1 *B*). The horizontal and vertical MFs, respectively, generate an external magnetic force parallel and perpendicular to the plane of expansion of yeast mats. The experimental and control devices were then placed inside an environmental chamber (Thermo Scientific, catalog no. 13-067-066) and incubated at 30°C and 50% humidity. Yeast mats seeded with TBR1 or TBR5 cells were grown for 25 days. The yeast mats were photographed daily using a Canon EOS Rebel SL3 camera with a Canon EF-S 3 mm f/2.8 Macro IS STM macro lens. The growth rates were evaluated from these photographs as described in agar culture growth rate measurements.

For the liquid culture horizontal and vertical MF exposure experiments, 24 5 mL culture tubes (Fisher Scientific, catalog no. 22-171-606; half containing TBR1 cells and half containing TBR5 cells) were placed inside four 3D-printed devices. Six TBR1 tubes and six TBR5 tubes were placed inside two separate experimental exposure devices. Another six TBR1 tubes and six TBR5 tubes were places inside two separate control devices. To maintain consistency with the agar culture-MF exposure experiments, the liquid culture-MF exposure experiments were also set up in the horizontal configuration. The experimental and control devices were then placed inside the environmental chamber on the shaker and incubated at 30°C and 50% humidity. The liquid TBR1 and TBR5 cultures were grown for 3 days. The growth rates were evaluated and the cultures re-suspended every 12 h (see liquid culture growth rate measurements).

### Agar culture growth rate measurements

Yeast mat area measurements were obtained daily for 25 days to determine the difference between the mat expansion rate of the control and the MF-exposed TBR1 and TBR5 strains. A quantitative analysis of the mat area expansion rates was performed using the image processing software ImageJ (40). The resolution of the original images was 6000 × 4000 pixels with an aspect ratio of 3:2. To obtain the area expansion rate from the original images, the original images were cropped to a square ratio (1:1) such that the circumference of the Petri dish in the image touched each of the four sides of the image. This enabled the accuracy to be maintained when scaling the image pixels by the length of the Petri dish in each image, as the area evaluation of the mats remained consistent throughout the analysis. After extracting the area of the mats from each image, the area expansion rates were obtained by dividing the total area at the end of each day by the number of days. Replicates were quickly removed, imaged, and placed back into the device one at time to minimize interference with the MF exposure; Petri dish lids were left on during imaging to reduce the risk of contamination.

### Liquid culture growth rate measurements

To determine the difference between the growth rates of the control and MF-exposed TBR1 and TBR5 strains in well-mixed YPD liquid cultures, cells were extracted from TBR1-seeded and TBR5-seeded mats using sterile 20 *μ*L pipette tips (Fisher Scientific, catalog no. 02-707-432). Specifically, 12 cultures of TBR1 cells were extracted from six TBR1 control and six TBR1 MF exposure plates and 12 cultures of TBR5 cells were extracted from six TBR5 control and six TBR5 MF exposure plates and grown in liquid YPD medium for 3 days. Every 12 h, cell counts were obtained using an automated cell counter (Corning, Corning, NY, USA, catalog no. C-6749), and the cell cultures were re-suspended to an initial concentration of $N_{0}=5.5\times 10^{5}$ cells/mL to keep them in log-phase growth. The population growth rate was calculated as follows (41):

$$r=\frac{1}{t_{r}}\ln\left(\frac{N\left(t\right)}{N_{0}}\right)\text{,}$$

where $t_{r}$ is the time interval between re-suspensions and N(t) is the cell count at time *t* after the re-suspension.

## Results

### MF device optimization and validation

The MF within the simulated exposure device had a well-defined orientation (Figs. 2 *A* and 3 *A*). Simulations of the vertical configuration of the exposure device produced $\vec{B}$ values ranging from 430 to 730 *G* along the horizontal surface of the central Petri dish (Fig. 2 *B*). The $\vec{B}$ values ranged from 500 to 1400 *G* along the central vertical plane of the exposure chamber (Fig. 2 *C*). Simulations of the horizontal configuration of the exposure device produced $\vec{B}$ values ranging from 500 to 1400 *G* (Fig. 3, *B* and *C*). In agreement with theory (36), the simulations predicted negligible interference with the MF when the exposure device is fabricated with materials with compatible *μ* values (Figs. 2 *A* and 3 *A*).

**Figure 4:**
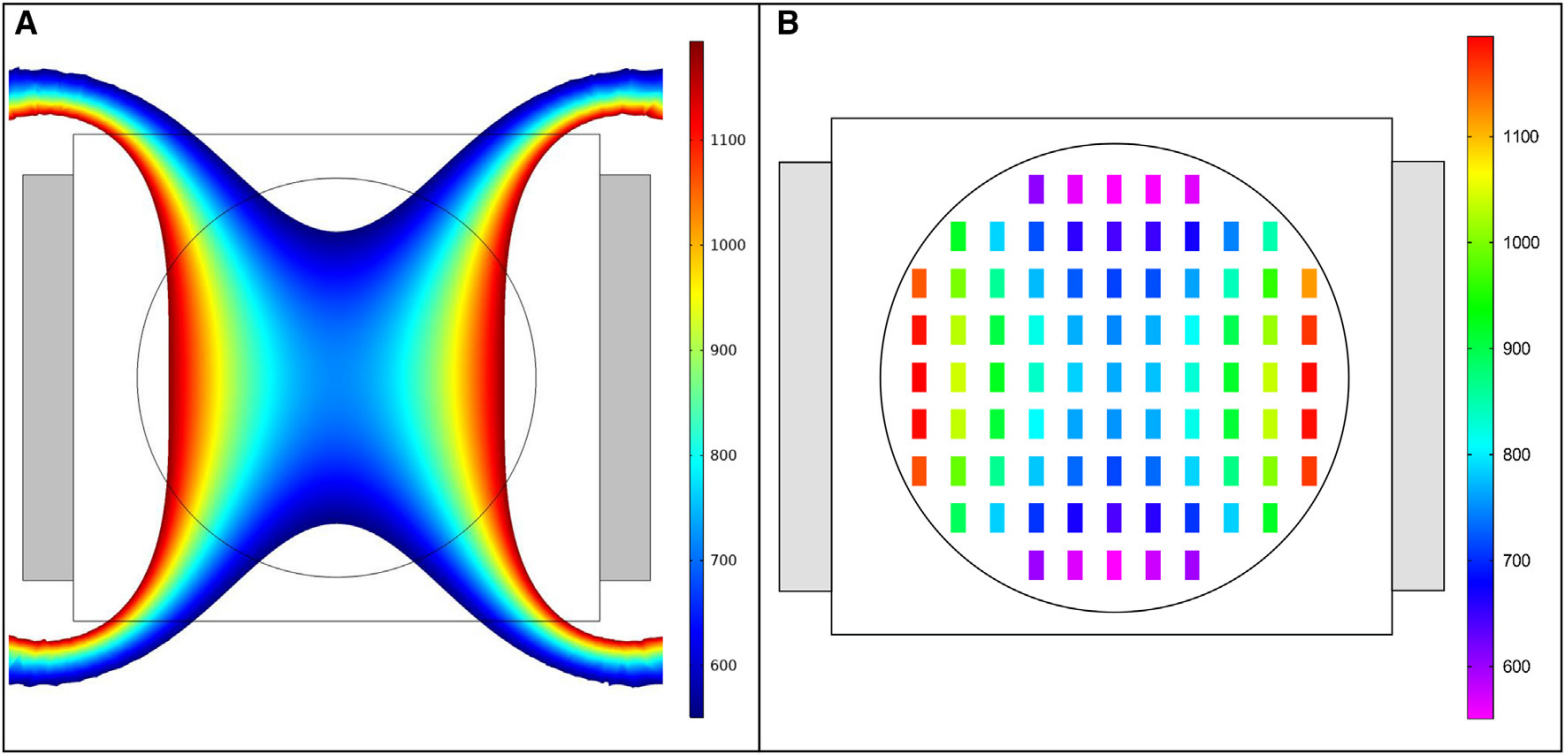
Comparison of simulation and experimental measurements of the horizontal configuration of the MF device. (*A*) Top view of a COMSOL (34) simulation of the $\vec{B}$ along the middle layer (layer 3) of the exposure chamber of the device. (*B*) Top view of the experimental $\vec{B}$ mapping of the sample exposure region of the device corresponding to (*A*). The gray blocks in (*A*) and (*B*) represent the neodymium magnets. The color bars denotes $\vec{B}$ in *G*.

Simulated and experimentally determined $\vec{B}$ maps for the middle layer (layer 3) of the horizontal configuration of the MF exposure device are shown in Fig. 4. The simulated $\vec{B}$ values for layer 3 ranged from 514.6 to 1147.5 *G* (Figs. 4 *A* and S3 *A*). The experimental $\vec{B}$ values for layer 3 ranged between 550.4 and 1194.4 *G* (Figs. 4 *B* and S3 *B*). The simulated and experimental $\vec{B}$ results for layers 1 and 2 are shown in Figs. S4 and S5, respectively. On average, each experimental $\vec{B}$ mapping value for layer 3 was 35.5 *G* different than the corresponding COMSOL simulated value (Fig. S6 *A*). This corresponds to a 4.4% average difference between the experimental and simulated $\vec{B}$ values (Fig. S6 *B*). Results for layers 1 and 2 follow a similar trend for the differences between the experimental and COMSOL-simulated $\vec{B}$ values. For layer 1, each experimental $\vec{B}$ value was 37.2 *G* less than the corresponding COMSOL-simulated value on average, which corresponds to a 5.7% difference between the experimental and simulated $\vec{B}$ values (Fig. S4). For layer 2, each experimental $\vec{B}$ value was 35.0 *G* less than the corresponding COMSOL-simulated value on average, which corresponds to a 4.5% difference between the experimental and simulated $\vec{B}$ values (Fig. S5). Overall, the experimental $\vec{B}$ measurements were in good agreement with the simulation results.

### Spatiotemporal yeast mat MF experiments

**Figure 5:**
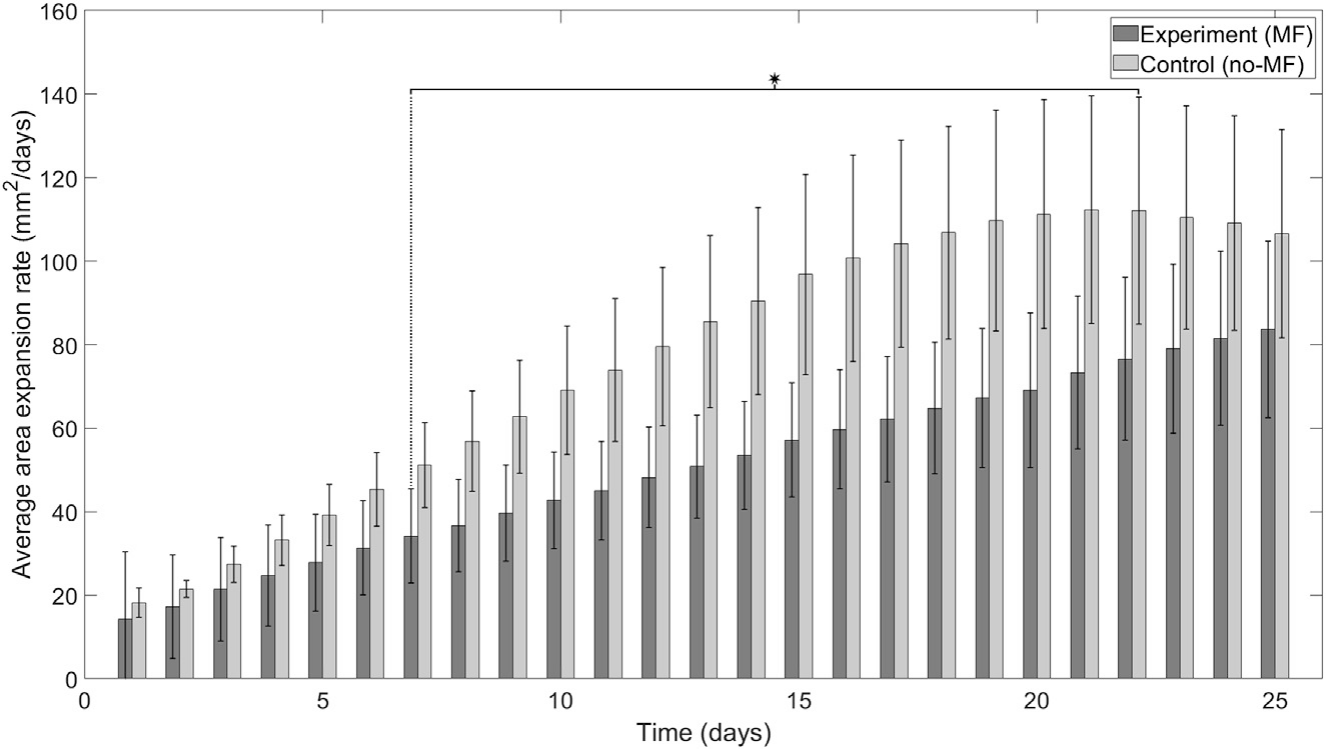
Average area expansion rates of TBR1 yeast mats in agar plates in the presence and absence of a horizontal MF. TBR1-seeded control (no-MF) and experimental (MF) mats grown on agar. An independent sample *t*-test was performed to compare the average expansion rates for a sample size of n=5. A significant difference in average area expansion rates independent sample *t* test (p<0.05, denoted by an asterisk) was found for days 7–22. Error bars denote standard deviation. For a Figure360 author presentation of this figure, see https://doi.org/10.1016/j.bpr.2024.100165.

**Figure 6:**
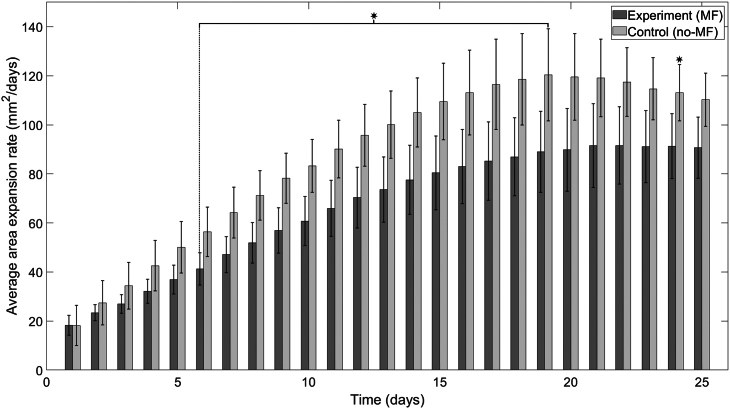
Average area expansion rates of TBR1 yeast mats in agar plates in the presence and absence of a vertical MF. TBR1-seeded control (no-MF) and experimental (MF) mats grown on agar. An independent sample *t*-test was performed to compare the average expansion rates for a sample size of n=4. A significant difference in average area expansion rates independent sample *t*-test (p<0.05, denoted by an asterisk) was found for days 6–19 and on day 24. Error bars denote standard deviation.

Horizontal and vertical MF exposure decreased the TBR1 mat area expansion rate (Figs. 5 and 6, respectively). Statistically significant differences in the average area expansion rate between the experimental (MF-exposed) and control (no-MF) groups occurred from day 7 to 22 for the horizontal MF exposure experiments (Figs. 5 and S7); the average area expansion rate of the TBR1 control group expanded at a linearly increasing rate (Table S2) until saturation around day 18 and then began to decrease. Statistically significant differences in the average area expansion rate between the experimental (MF-exposed) and control (no-MF) groups occurred from day 6 to 19 and on day 24 for the vertical MF exposure experiments (Figs. 6 and S8); the average area expansion rate of the control group expanded at a linearly increasing rate (Table S3) until saturation around day 15 and then began to decrease. The saturation and subsequent decrease in the average area expansion rates of the faster growing control groups can be attributed to nutrient depletion as the yeast mats expand across the agar surface (29,42). The average area expansion rate of the horizontal MF-exposed group displayed a slower, monotonic, and linearly increasing growth (Table S2). The average area expansion rate of the vertical MF-exposed group also displayed a slower, monotonic, and linearly increasing growth until saturation around day 17 (Table S3). The average area of the control and horizontal MF-exposed TBR1 mats increased exponentially (Table S4). The average area of the control and vertical MF-exposed TBR1 mats increased linearly (Table S5).

**Figure 7:**
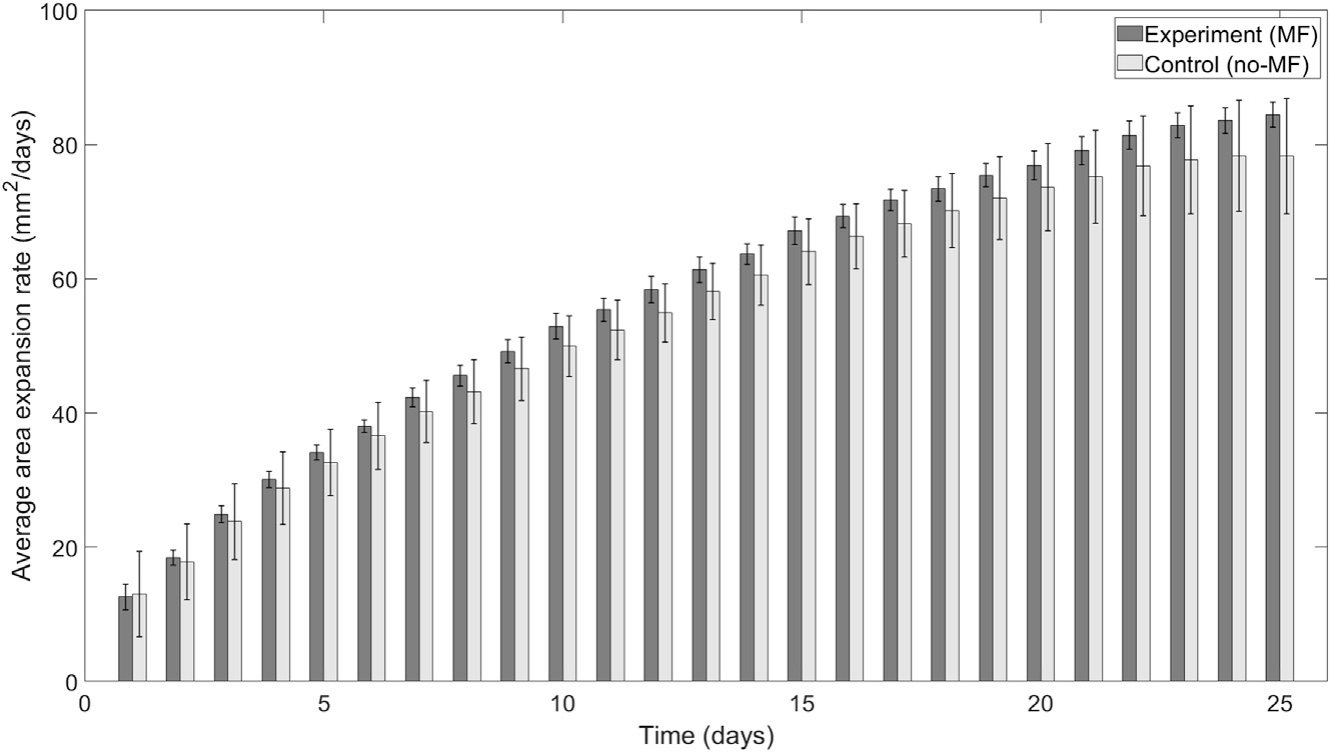
Average area expansion rates of TBR5 yeast mats in the presence and absence of a horizontal MF. TBR5-seeded control (no-MF) and experimental (MF) mats grown on agar plates. An independent sample *t*-test was performed to compare the average expansion rates for a sample size of n=3. No significant difference in average area expansion rates (p>0.05) was found for any day. Error bars denote standard deviation.

**Figure 8:**
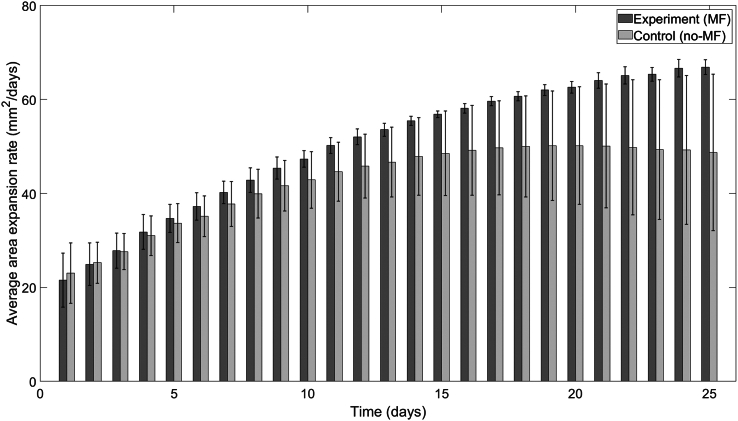
Average area expansion rates of TBR5 yeast mats in the presence and absence of a vertical MF. TBR5-seeded control (no-MF) and experimental (MF) mats grown on agar plates. An independent sample *t*-test was performed to compare the average expansion rates for a sample size of n=4. No significant difference in average area expansion rates (p>0.05) was found for any day. Error bars denote standard deviation.

Horizontal and vertical MF exposure did not impact the TBR5 mat area expansion rate (Figs. 7 and S9 and Figs. 8 and S10, respectively). Since the TBR5 expansion rate is slower compared to TBR1, the TBR5 average area expansion rates did not saturate during the experiment (Figs. 7 and 8, respectively). This likely can be attributed to TBR5 mats not sufficiently depleting the nutrients in the agar media. Experimental and control groups of TBR5 yeast mats for the horizontal and vertical MF-experiments displayed a logarithmic average area expansion rates (Tables S2 and S3, respectively), which increased monotonically for the duration of the experiment. The average area of control and MF-exposed TBR5 mats for the horizontal and vertical MF-experiments expanded linearly (Tables S4 and S5).

### Well-mixed planktonic yeast MF experiments

**Figure 9:**
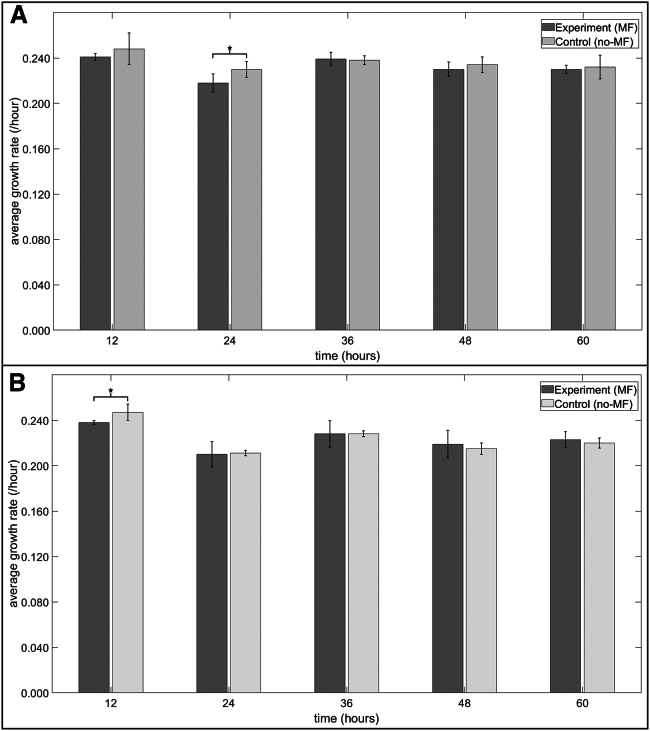
Average growth rates of TBR1 and TBR5 cells in liquid culture in the presence and absence of a horizontal MF. (*A*) Average growth rates of TBR1. An independent sample *t*-test was performed to compare the average growth rates between the exposure (MF) and control (no-MF) replicates for a sample size of n=6. A significant difference (p<0.05) was only observed at the 24 h time point. (*B*) Average growth rate of TBR5. An independent sample *t*-test was performed to compare the average growth rates between the exposure (MF) and control (no-MF) replicates for a sample size of N=6. A significant difference (p<0.05) was only observed at the 12 h time point. Error bars denote standard deviation.

**Figure 10:**
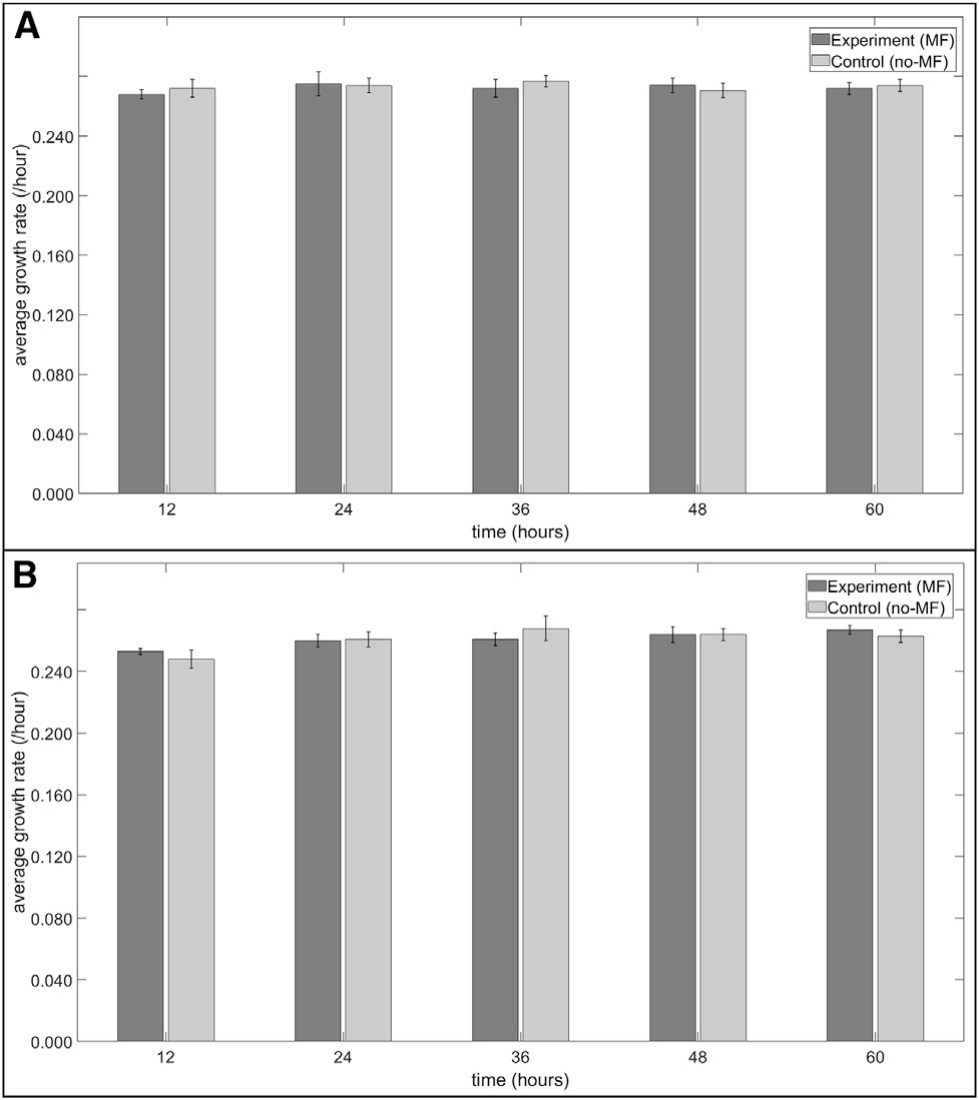
Average growth rates of TBR1 and TBR5 cells in liquid culture in the presence and absence of a vertical MF. (*A*) Average growth rates of TBR1. (*B*) Average growth rates of TBR5. For (*A*) and (*B*), an independent sample *t*-test was performed to compare the average growth rates between the exposure (MF) and control (no-MF) replicates for a sample size of n=6; no significant difference (p>0.05) was observed at any time point. Error bars denote standard deviation.

Overall, horizontal and vertical MF exposure did not affect the steady-state growth of TBR1 and TBR5 cells cultured in liquid media (Figs. 9 and 10). No significant differences were observed in TBR1 average growth rates between the control and horizontal MF-exposed groups at the 12, 36, 48, and 60 h time points (Fig. 9 *A*), and no significant differences were observed in TBR5 average growth rates between horizontal MF-exposed and control groups at the 24, 36, 48, and 60 h time points (Fig. 9 *B*). Vertical MF exposure did not affect the steady-state growth of TBR1 and TBR5 cells cultured in liquid media (Fig. 10). No significant differences were observed in TBR1 and TBR5 average growth rates between the control and MF-exposed groups at any time points. The similarity in the growth rates of TBR1 and TBR5 can likely be explained by the negation of the function of the *flo11* gene in liquid media. These results are in agreement with previous TBR1 and TBR5 liquid culture experiments (31,32).

## Discussion

In this study, we developed an open-source MF exposure platform to perform magnetobiological experiments on well-mixed and spatially structured populations of microorganisms. Our 3D-printed device was designed in AutoCAD (33) to have interchangeable horizontal and vertical MF configurations and hold multiple culture tubes and Petri dishes. The strength, size, and position of the magnets were optimized for MF exposure experiments on the budding yeast *S. cerevisiae* using numerical simulations in COMSOL (34) together with Gaussmeter measurements. The AutoCAD, COMSOL, and 3D printing files are freely available for use in other MF experiments (see supporting material). In contrast to previous experimental work that focused on individual yeast cells (12), our MF device provides the ability to study the effects of MFs on populations of yeast cells. By using two large neodymium magnets, the exposure chamber is sufficiently large enough to expose yeast mats/biofilms on agar plates and yeast cells in liquid culture to a homogeneous MF. Additionally, while previous MF exposure experiments were conducted for no longer than 2 days (13,14,15,12), our MF exposure device is able to maintain an uninterrupted MF for longer-term experiments, the importance of which has been previously emphasized (20). Our compact device can be placed inside of a standard microbiological incubator or environmental chamber to control for confounding environmental variables, such as temperature, that affect growth and gene expression in yeast (43). We note that our MF device can be used to explore MF effects on a microorganisms beyond yeast, for instance, planktonic bacteria and bacterial biofilms.

We used our MF exposure device to investigate the effects of MFs on the growth of two genetically engineered *S. cerevisiae* strains in both agar and liquid media. We discovered that horizontal and vertical MFs slowed the spatially structured expansion of TBR1 yeast mats on agar but did not affect the expansion of TBR5 yeast mats. We also found that vertical and horizontal MFs did not affect the growth of well-mixed TBR1 and TBR5 yeast cells in liquid media. We hypothesize that the decreased expansion rate of horizontal MF-exposed TBR1 yeast mats results from spatial hindrance in the 2D expansion of this strain (31) combined with the magnetic properties of microtubules (12,44,45). As the mitotic spindle is composed of (46) and oriented by (47) microtubules, the presence of a horizontal MF may cause the budding yeast cells to align their long axis along the direction of the MF (20,12). Partial or complete alignment of cells to the MF at the expanding boundary of a yeast mat may introduce competition among cells attempting to bud into unoccupied space on the agar surface. These experimental results qualitatively agree with computational predictions (20). Related demography-dispersal trade-off effects have recently been reported in competing and evolving TBR1 and TBR5 yeast mats in the absence of MFs (48). As for vertical MF exposure, we hypothesize that the magnetic force negates the surface adhesion of *Flo11*, which allows TBR1 to expand in 3D (similar to TBR5), leading to slower radial expansion across the agar surface. MF-induced steric hindrance may also explain the previously observed slowed growth of phytopathogenic fungi exposed to MFs (17); increased cell-to-cell contact forces resulting from MF alignment may destabilize polarized growth machinery (49). While TBR5 cells would experience similar MF orientation effects to TBR1 cells, the fact that TBR5 mats expand in 3D across the agar surface may reduce spatial hindrance effects at the expanding mat front. No MF-related effects were observed when planktonic TBR1 and TBR5 strains were grown in liquid media. This can likely be attributed to negation of *flo11* in well-mixed environments, rendering the fitness of planktonic TBR1 and TBR5 cells equal.

Previous studies have highlighted the importance of studying the nonlinear effects of MF on biological systems at specific flux densities (e.g., (6)). As our device was designed to expose microbial populations to a range of flux density values, the current design lacks the ability to generate a single MF strength throughout the exposure region. Although we simulated and experimentally mapped the flux density values at various locations within the exposure region, we did not evaluate the biological effects of MFs at specific flux density values. Such studies may be performed by adapting our MF exposure platform to incorporate a Gaussmeter probe and multiple cameras or a stage-mounted camera. We also note that studying the effects of inhomogeneous MFs on microbial populations may prove to be a promising line of future research. To facilitate this research, smaller magnets could be used with our device to expose microbiological samples to MF edge effects. MF effects at the single-cell level (12) may be important to elucidate the mechanisms underlying our population-scale experimental results. Our MF device could be augmented with a camera for microscopic imaging to study MF effects in real time at the single-cell level in microbial populations. Other future improvements to our MF device include using a 3D-printed material other than PLA (which has rigidity at temperatures between 45°C and 60°C (50)), such as nylon or carbon fiber that can withstand the high temperature and pressure of an autoclave (51). Finally, performing experiments with many exposure devices in parallel will ensure sufficient biological replicates with identical vertical MF conditions.

It will be imperative to elucidate the biophysical mechanisms underlying our experimental results. The magnetization of microtubules should be investigated in the context of yeast mat and biofilm development. Previous studies have reported magnetic properties of microtubules (45,52), and it has been proposed that the polarization of microtubules is responsible for the alignment of *S. cerevisiae* with MFs (12). As radical pairs may play a role in microtubule reorganization, this should also be investigated as a possible mechanism underlying magnetic phenomena in yeast (53). Overall, we anticipate that our MF exposure device and experimental findings will advance our fundamental understanding of magnetic phenomena in microbes.

## Author contributions

D.A.C. conceptualized the study. A.B. designed, modeled, built, and validated the MF device. A.B. performed the yeast experiments with assistance from E.L. A.B. visualized and analyzed the results. D.A.C. and A.B. wrote the manuscript. D.C. supervised the study.

## Supporting material

Document S1. Figures S1–S10 and Tables S1–S5

Figure 360. An author presentation of Figure 5

Document S2. Article plus supporting material
