# Supplementary material for: Magnetic field platform for experiments on well-mixed and spatially structured microbial populations
Source: Biophys Rep (N Y). 2024 Jun 17;4(3):100165. doi: 10.1016/j.bpr.2024.100165 (PMC11276921; doi:10.1016/j.bpr.2024.100165)
Supplement: Document S1. Figures S1–S10 and Tables S1–S5 [file mmc1.pdf]

Biophysical Reports, Volume *4*

Supplemental information

Magnetic field platform for experiments on well-mixed and spatially structured microbial populations

Akila Bandara, Enoki Li, and Daniel A. Charlebois

APPENDICES

Supplemental Tables

Parameter	Value
Permeability of Vacuum (μ_0)	$4\pi \times 10^7\ Hm^{-1}$
Relative Permeability of Air ($\mu_{r,air}$)	1.00
Relative Permeability of PLA ($\mu_{r,PLA}$)	1.00
Relative Permeability of Petri Dishes ($\mu_{r,Petri}$)	1.00
Recoil Permeability of N52 grade $Nd_2Fe_{14}B$ ($\mu_{rec,N52}$)	1.05
Remnant flux density of N52 grade $Nd_2Fe_{14}B$ (B_r)	14400 G

Table S1: Parameter values used in the COMSOL simulation of the magnetic field exposure device. The values for the N52 grade $Nd_2Fe_{14}B$ magnets were obtained from the COMSOL material library [1].

Parameters	Curve Fit	TBR1 Exposed	TBR1 Control	TBR5 Exposed	TBR5 Control
SSE	Linear	**15.806**	**45.509**	411.219	395.784
	Exponential	435.510	781.309	1255.700	1150.900
	Logarithmic	1306.600	1728.500	**72.755**	**66.278**
R^2	Linear	**0.999**	**0.997**	0.964	0.960
	Exponential	0.959	0.947	0.889	0.883
	Logarithmic	0.878	0.883	**0.994**	**0.993**
$AdjR^2$	Linear	**0.999**	**0.997**	0.962	0.958
	Exponential	0.957	0.944	0.884	0.878
	Logarithmic	0.872	0.876	**0.993**	**0.993**
$RMSE$	Linear	**0.829**	**1.687**	4.228	4.148
	Exponential	4.351	6.988	7.389	7.074
	Logarithmic	7.537	10.394	**1.779**	**1.698**

Table S2: Goodness of fit of the average area expansion rate data for TBR1-TBR5 control (no MF) and experimental (MF) group data for the horizontal MF experiments. The model (linear, exponential, and logarithmic) with the best goodness of fit statistic are highlighted in green for TBR1 and yellow for TBR5. See main text for details on days over which fits were performed.

Parameters	Curve Fit	TBR1 Exposed	TBR1 Control	TBR5 Exposed	TBR5 Control
SSE	Linear	**58.579**	**90.219**	234.983	333.159
	Exponential	465.682	795.048	516.109	42235.000
	Logarithmic	758.628	920.929	**29.074**	**87.353**
R^2	Linear	**0.993**	**0.993**	0.950	0.815
	Exponential	0.941	0.934	0.891	-22.458
	Logarithmic	0.903	0.924	**0.994**	**0.952**
$Adj R^2$	Linear	**0.992**	**0.992**	0.948	0.807
	Exponential	0.937	0.929	0.886	-23.478
	Logarithmic	0.897	0.918	**0.994**	**0.949**
$RMSE$	Linear	**1.975**	**2.634**	3.196	3.806
	Exponential	5.572	7.820	4.737	42.852
	Logarithmic	7.112	8.417	**1.124**	**1.949**

Table S3: Goodness of fit of the average area expansion rate data for TBR1-TBR5 control (no MF) and experimental (MF) group data for vertical MF experiments. The model (linear, exponential, and logarithmic) with the best goodness of fit statistic are highlighted in green for TBR1 and yellow for TBR5. See main text for details on days over which fits were performed.

Parameters	Curve Fit	TBR1 Exposed	TBR1 Control	TBR5 Exposed	TBR5 Control
SSE	Linear	405950.0	252690.0	**195520.0**	**159100.0**
	Exponential	**227490.0**	**192420.0**	420520.0	414560.0
	Logarithmic	3010800.0	1842900.0	2691900.0	2340400.0
R^2	Linear	0.961	0.963	**0.982**	**0.984**
	Exponential	**0.978**	**0.972**	0.962	0.958
	Logarithmic	0.707	0.728	0.756	0.760
$Adj R^2$	Linear	0.959	0.960	**0.982**	**0.983**
	Exponential	**0.977**	**0.970**	0.960	0.956
	Logarithmic	0.695	0.711	0.746	0.750
$RMSE$	Linear	132.853	125.670	**92.201**	**93.170**
	Exponential	**99.452**	**109.665**	135.216	134.255
	Logarithmic	361.805	339.385	342.113	318.990

Table S4: Goodness of fit of average area data for TBR1-TBR5 control (no MF) and experimental (MF) group data for the horizontal MF experiments. The model (linear, exponential, and logarithmic) with the best goodness of fit statistic are highlighted in green for TBR1 and yellow for TBR5. See main text for details on days over which fits were performed.

**Parameters**	**Curve Fit**	**TBR1 Exposed**	**TBR1 Control**	**TBR5 Exposed**	**TBR5 Control**
SSE	Linear	**99817.0**	**119510.0**	**64516.0**	**10779.0**
	Exponential	116070.0	119520.0	286630.0	254600.0
	Logarithmic	891260.0	985510.0	1457000.0	617280.0
R^2	Linear	**0.972**	**0.971**	**0.990**	**0.997**
	Exponential	0.968	0.971	0.957	0.931
	Logarithmic	0.753	0.758	0.783	0.833
$AdjR^2$	Linear	**0.970**	**0.968**	**0.990**	**0.997**
	Exponential	0.966	0.968	0.956	0.928
	Logarithmic	0.736	0.739	0.774	0.826
$RMSE$	Linear	**81.575**	**95.879**	**52.963**	**21.648**
	Exponential	87.965	95.885	111.634	105.212
	Logarithmic	243.757	275.333	251.691	163.823

Table S5: Goodness of fit of average area data for TBR1-TBR5 control (no MF) and experimental (MF) group data for the vertical MF experiments. The model (linear, exponential, and logarithmic) with the best goodness of fit statistic are highlighted in green for TBR1 and yellow for TBR5. See main text for details on days over which fits were performed.

The following statistics were used to evaluate the goodness of fit for the data in Tables S2-S5. The SSE - sum of squares due to error:

$$SSE = \sum_{i=1}^{n} (y_i - \hat{y}_i)^2;$$

R^2 - ratio between the sum of squares of the regression (SSR) and the total sum of squares (SST):

$$R^2 = \frac{SSR}{SST} = \frac{\sum_{i=1}^{n} (\hat{y}_i - \bar{y})^2}{\sum_{i=1}^{n} (y_i - \bar{y})^2} = 1 - \frac{SSE}{SST} = 1 - \frac{\sum_{i=1}^{n} (y_i - \hat{y}_i)^2}{\sum_{i=1}^{n} (y_i - \bar{y})^2};$$

$AdjR^2$ - degrees of freedom adjusted R^2:

$$AdjR^2 = 1 - \frac{SSE(n-1)}{SST(\nu)} = 1 - \frac{\sum_{i=1}^{n} (y_i - \hat{y}_i)^2 (n-1)}{\sum_{i=1}^{n} (y_i - \bar{y})^2 (\nu)};$$

and $RMSE$ - root mean squared error:

$$RMSE = \sqrt{\frac{SSE}{\nu}} = \sqrt{\frac{\sum_{i=1}^{n} (y_i - \hat{y}_i)^2}{\nu}}.$$

For the above equations, y_i is the ith value of the variable to be predicted, $\hat{y}_i$ the predicted value of y_i, $\bar{y}$ the mean of all values of y_i, n the number of data points, ν the number of number of degrees of freedom, and $(\nu = n - m)$, where m is the number of fitted coefficients estimated from the data points.

Supplemental Figures

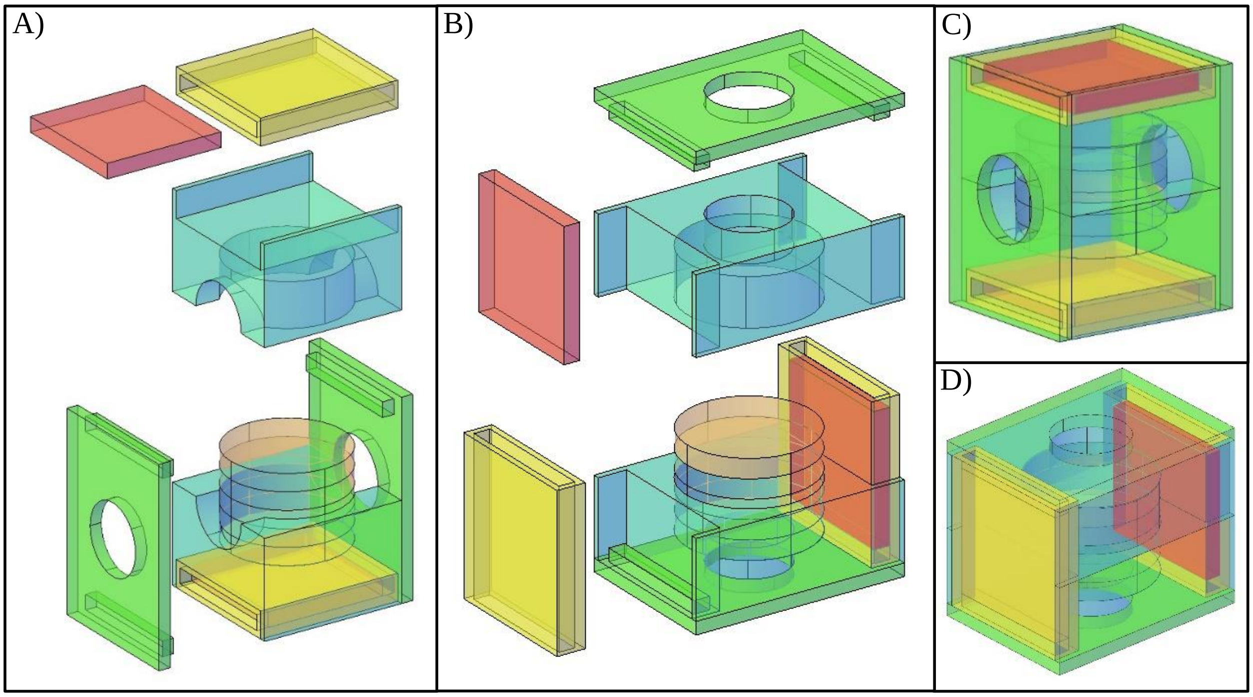

Figure S1: Modular design of the magnetic field exposure device. (A) AutoCAD [2] image of the disassembled vertical magnetic field (MF) configuration of the device. (B) AutoCAD image of the disassembled horizontal MF configuration of the device. (C) Assembled AutoCAD image of the vertical MF configuration of the device. (D) Assembled AutoCAD image of the horizontal MF configuration of the device. The magnets are depicted in red, magnet holders in yellow, Petri dish holders in cyan, Petri dishes in orange, and the yokes (parts that hold the device together after assembly) in green.

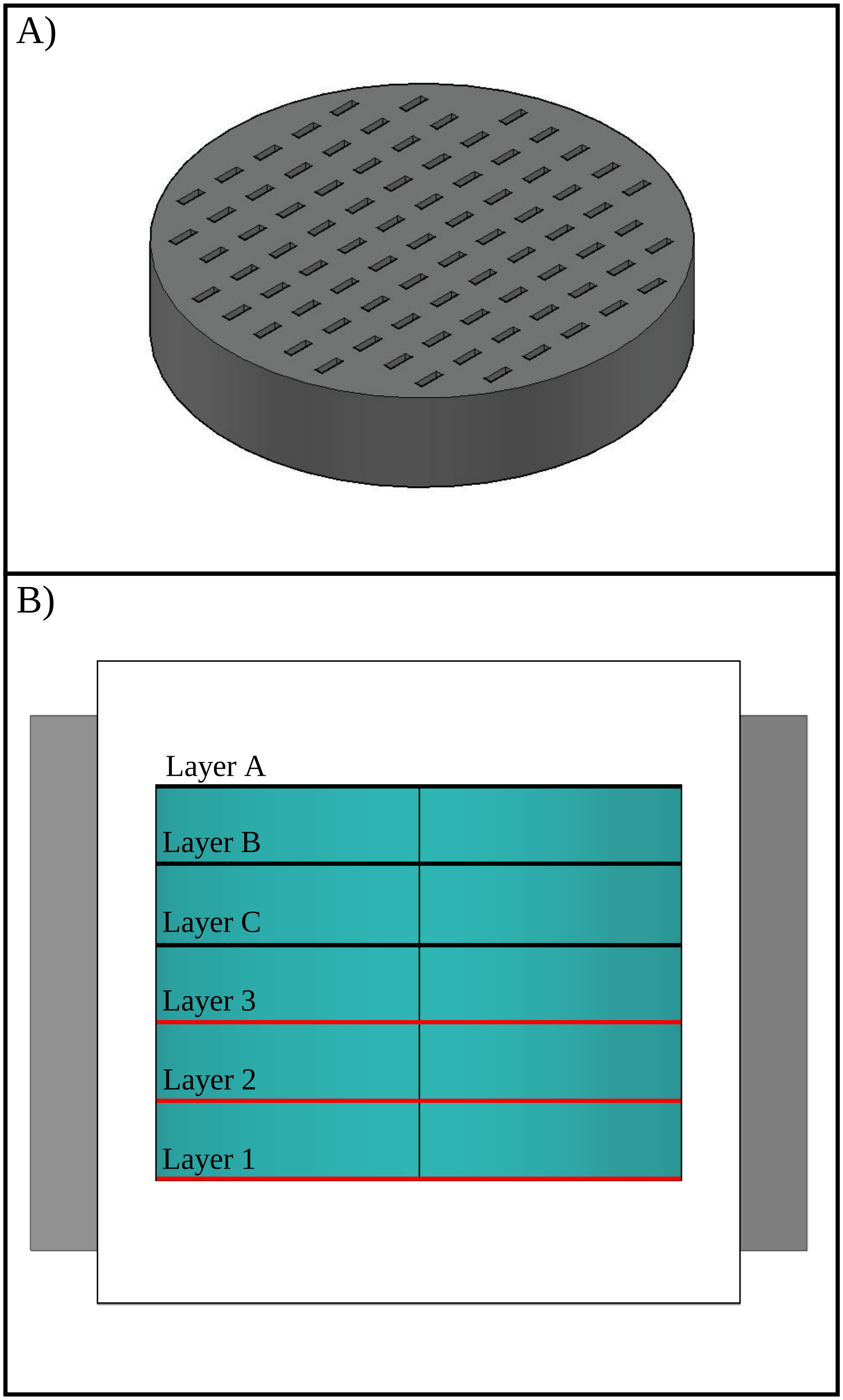

Figure S2: Experimental setup to map the magnetic flux density ($\vec{B}$). (A) AutoCAD [2] image of the cylindrical device with 83 rectangular holes used to hold the Gaussmeter probe during $\vec{B}$ measurements. (B) Schematic of three different layers in which $\vec{B}$ was mapped using the Gaussmeter. The grey blocks denote the permanent magnets and the Petri dishes are shown in blue. The red line indicate the layers that were evaluated in the $\vec{B}$ mapping.

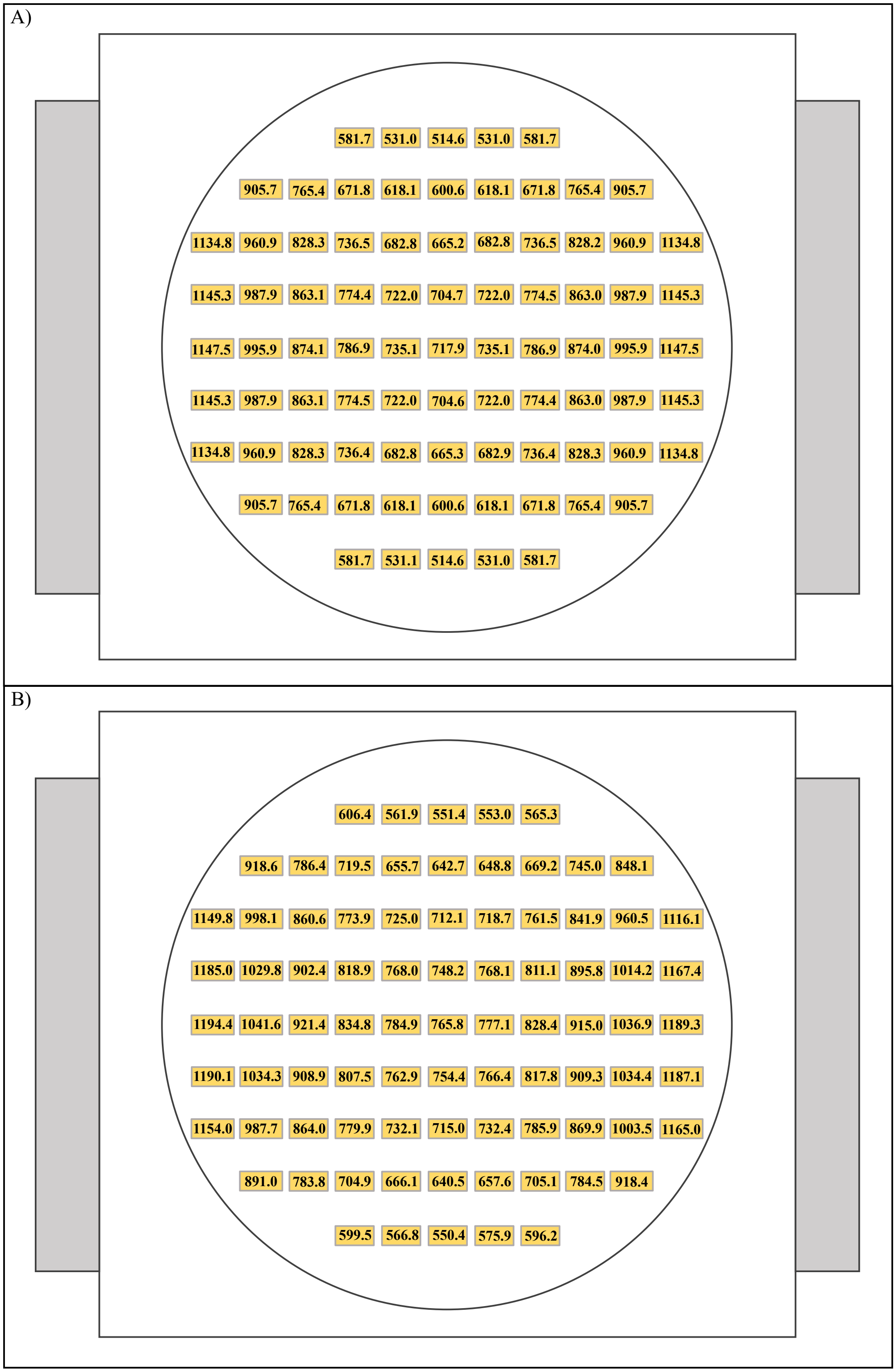

Figure S3: Simulated and experimentally measured values of the magnetic flux density of the horizontal configuration of the magnetic field device for layer 3. The point of view for this figure is from the top view of the middle layer (layer 3). (A) Magnetic flux density ($\vec{B}$) values obtained from a COMSOL [1] simulation. (B) $\vec{B}$ values obtained from Gaussmeter measurements. Values of $\vec{B}$ in (A) and (B) are in Gauss (G).

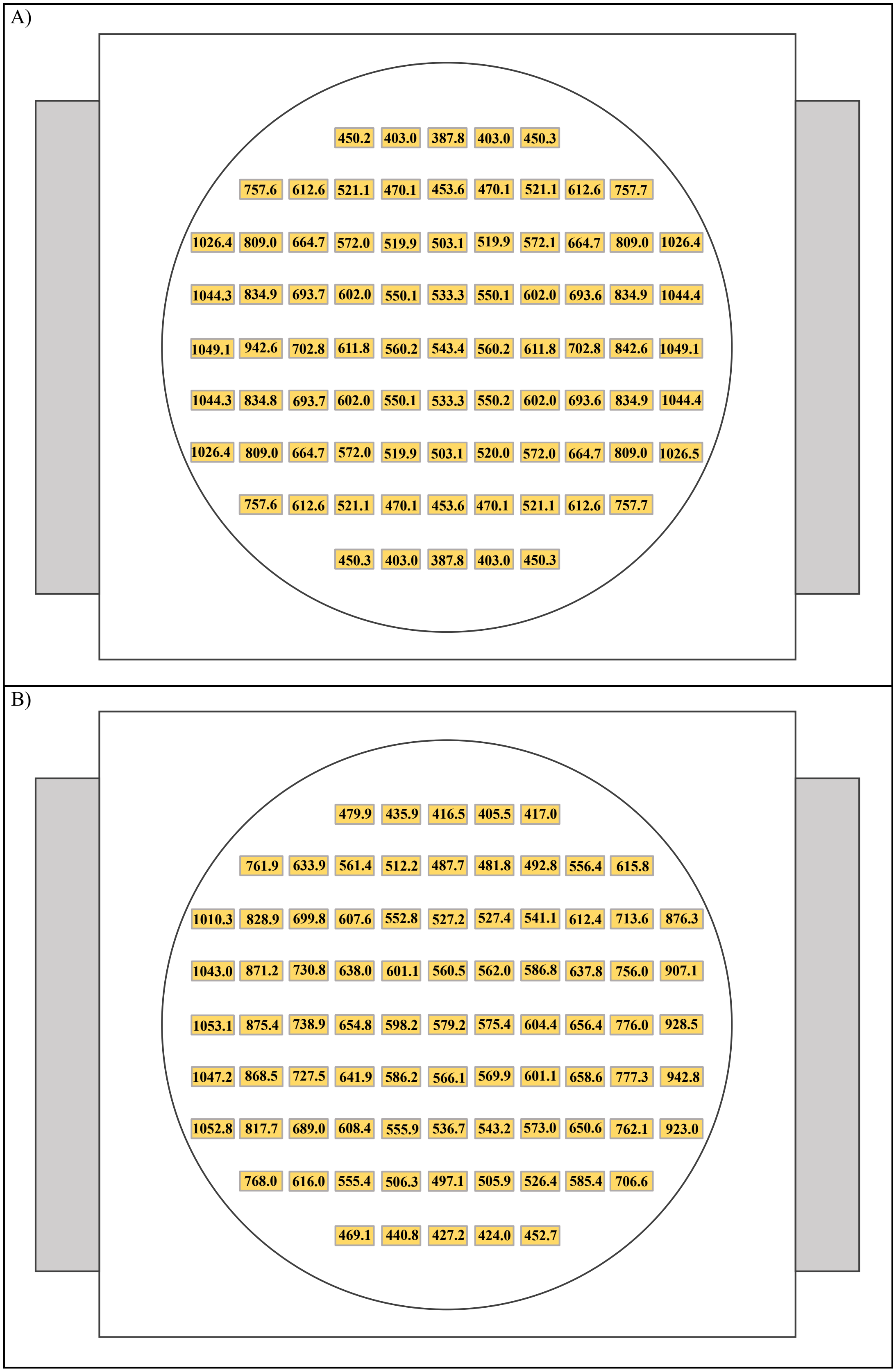

Figure S4: Simulated and experimentally measured values of the magnetic flux density of the horizontal configuration of the magnetic field device for layer 1. The point of view for this figure is from the top view of the layer 1. (A) Magnetic flux density ($\vec{B}$) values obtained from a COMSOL [1] simulation. (B) $\vec{B}$ values obtained from Gaussmeter measurements. Values of $\vec{B}$ in (A) and (B) are in Gauss (G).

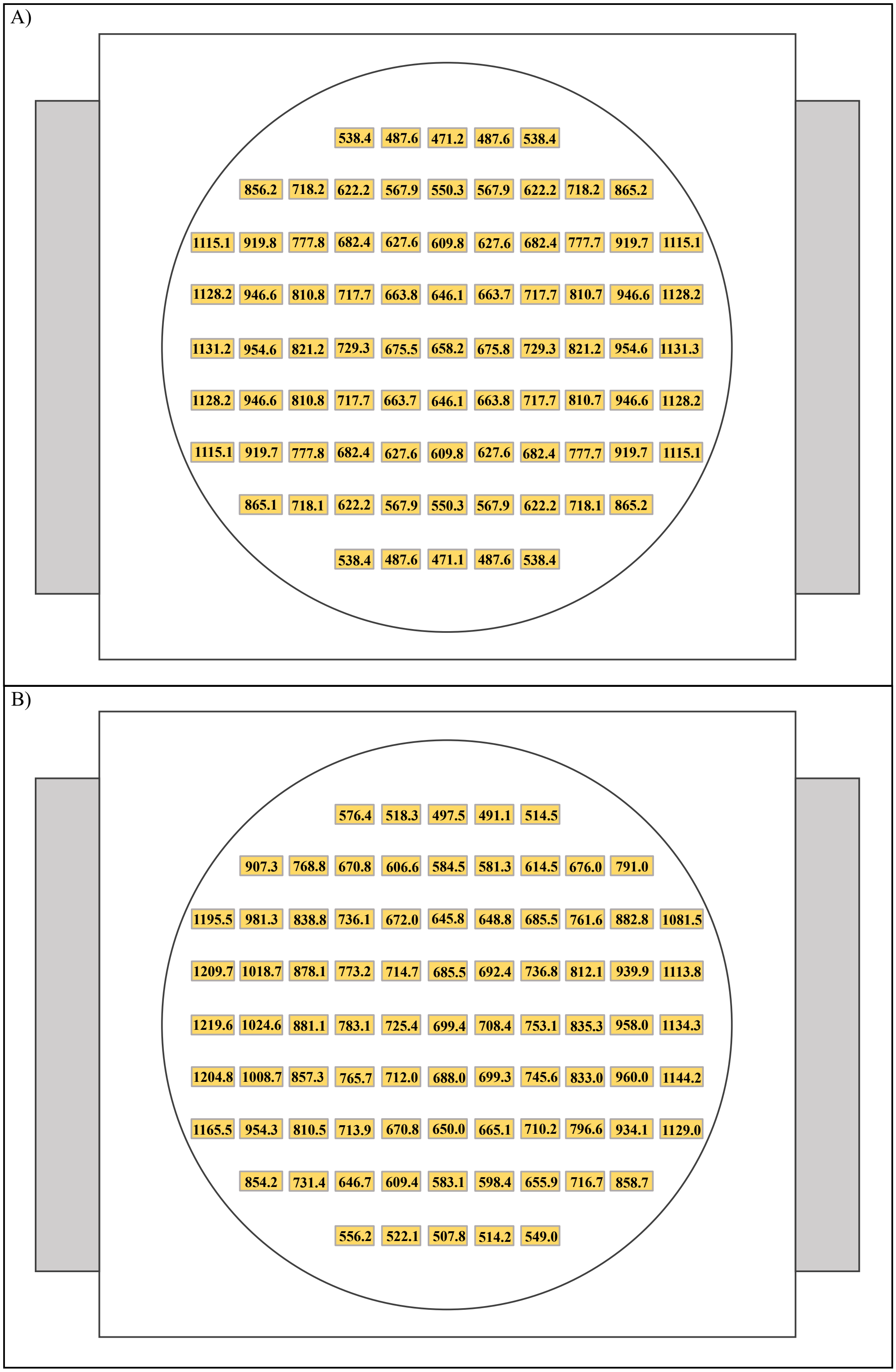

Figure S5: Simulated and experimentally measured values of the magnetic flux density of the horizontal configuration of the magnetic field device for layer 2. The point of view for this figure is from the top view of the layer 2. (A) Magnetic flux density ($\vec{B}$) values obtained from a COMSOL [1] simulation. (B) $\vec{B}$ values obtained from Gaussmeter measurements. Values of $\vec{B}$ in (A) and (B) are in Gauss (G).

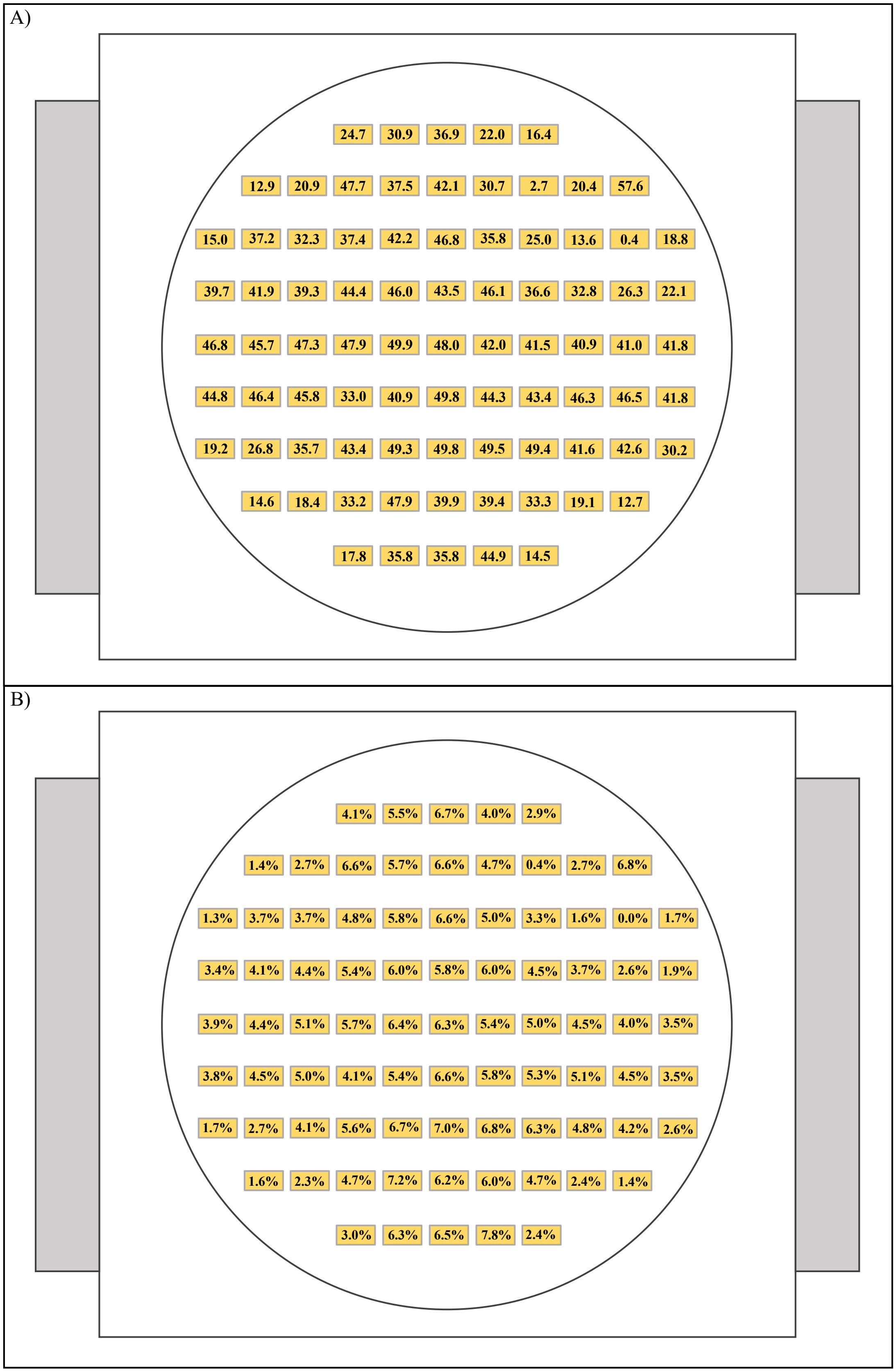

Figure S6: Difference between simulated and experimental magnetic flux densities of horizontal configuration of the magnetic field device for layer 3. The point of view for this figure is from the top view of the middle layer (layer 3). (A) The difference between the simulated and measured magnetic flux densities ($\vec{B}$). Values of $\vec{B}$ are in Gauss (G). (B) The difference between the simulated and experimentally measured $\vec{B}$ values as a percentage of the experimental values.

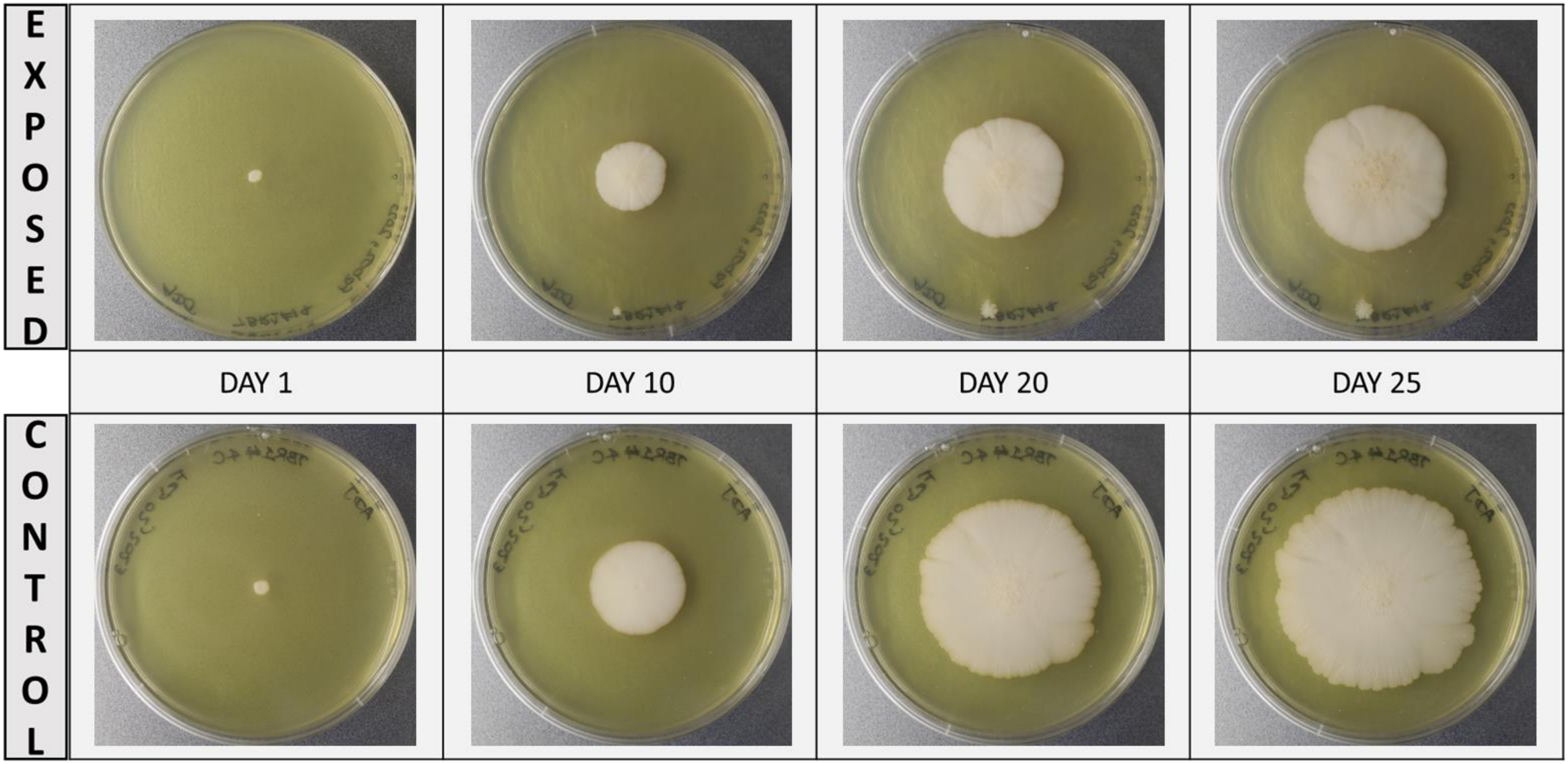

Figure S7: Representative images of the development of TBR1 yeast mats for (Top) the horizontal MF exposed condition and for (Bottom) the control condition (no MF).

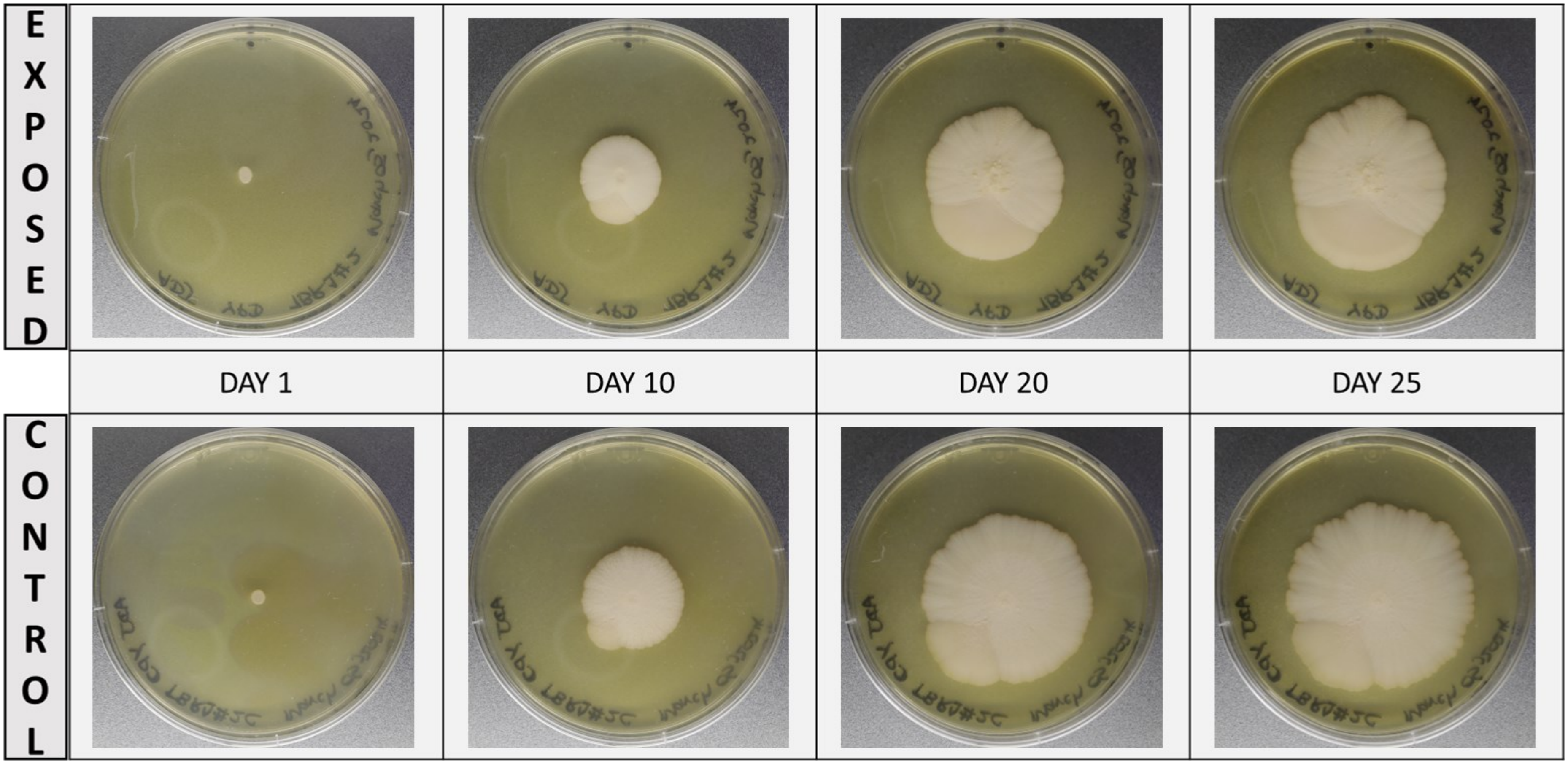

Figure S8: Representative images of the development of TBR1 yeast mats for (Top) the vertical MF exposed condition and for (Bottom) the control condition (no MF).

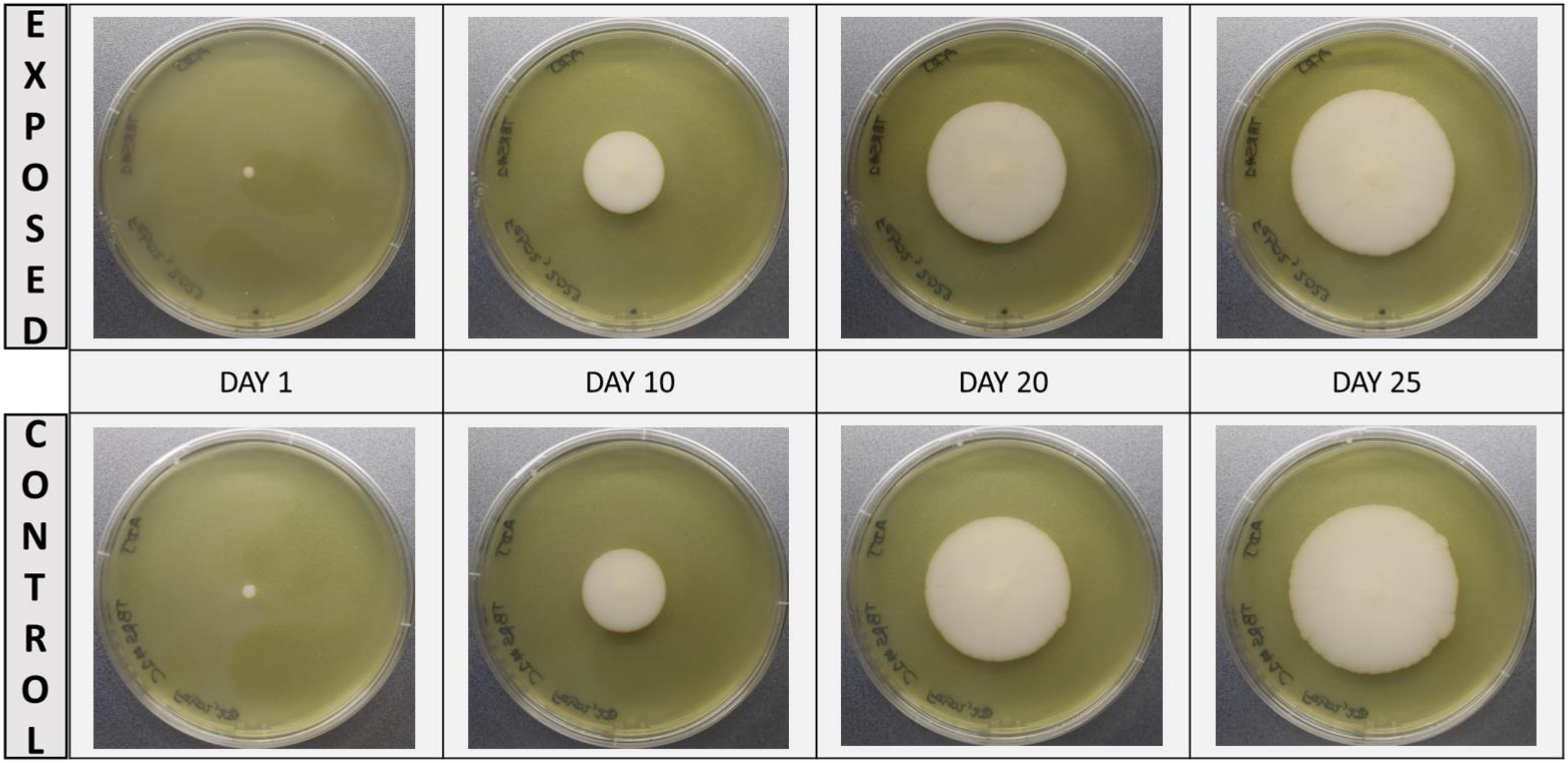

Figure S9: Representative images of the development of TBR5 yeast mats for (Top) the horizontal MF exposed condition and for (Bottom) the control condition (no MF).

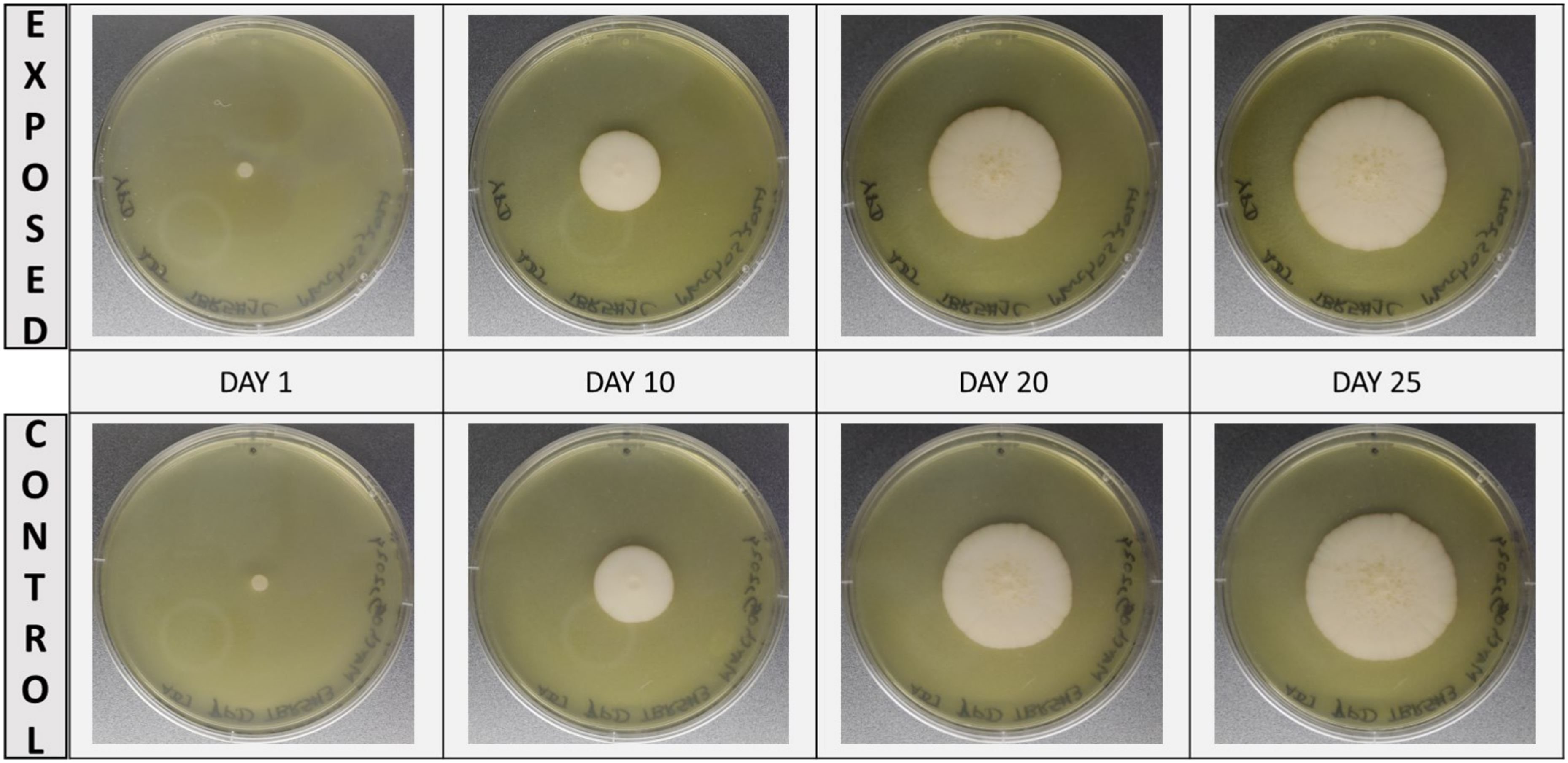

Figure S10: Representative images of the development of TBR5 yeast mats for (Top) the vertical MF exposed condition and for (Bottom) the control condition (no MF).

References

[1] COMSOL Inc. COMSOL Multiphysics. https://www.comsol.com/comsol-multiphysics, Version - 6.0.

[2] 2021 Autodesk Inc. AutoCAD 2022. https://manage.autodesk.com/products, Version - S.51.0.0 AutoCAD 2022.
